# Hydroxyacetophenone defenses in white spruce against spruce budworm

**DOI:** 10.1111/eva.12885

**Published:** 2019-12-20

**Authors:** Geneviève J. Parent, Claudia Méndez‐Espinoza, Isabelle Giguère, Melissa H. Mageroy, Martin Charest, Éric Bauce, Joerg Bohlmann, John J. MacKay

**Affiliations:** ^1^ Département des sciences du bois et de la forêt Centre d’étude de la forêt Université Laval Québec QC Canada; ^2^ Institut de biologie intégrative et des systèmes Université Laval Québec QC Canada; ^3^ Department of Plant Sciences University of Oxford Oxford UK; ^4^ Centro Nacional de Investigación Disciplinaria en Conservación y Mejoramiento de Ecosistemas Forestales Instituto Nacional de Investigaciones Forestales, Agrícolas y Pecuarias Ciudad de México Mexico; ^5^ Michael Smith Laboratories University of British Columbia Vancouver BC Canada; ^6^ Norwegian Institute for Bioeconomy Research Ås Norway

**Keywords:** chemical defense, forestry, herbivory, quantitative genetics, species interactions, transcriptomics

## Abstract

We review a recently discovered white spruce (*Picea glauca*) chemical defense against spruce budworm (*Choristoneura fumiferana)* involving hydroxyacetophenones. These defense metabolites detected in the foliage accumulate variably as the aglycons, piceol and pungenol, or the corresponding glucosides, picein and pungenin. We summarize current knowledge of the genetic, genomic, molecular, and biochemical underpinnings of this defense and its effects on *C. fumiferana*. We present an update with new results on the ontogenic variation and the phenological window of this defense, including analysis of transcript responses in *P. glauca* to *C. fumiferana* herbivory. We also discuss this chemical defense from an evolutionary and a breeding context.

## 1 INTRODUCTION

A chemical defense against spruce budworm (*Choristoneura fumiferana* (Clemens)) involving the accumulation of hydroxyacetophenones, specifically piceol (4‐hydroxyacetophenone) and pungenol (3,4‐dihydroxyacetophenone), was recently characterized in white spruce (*Picea glauca* (Moench) Voss, Mageroy et al., 2015). Hydroxyacetophenone aglycons and expression of the gene responsible for their release from the corresponding glucosides were identified as potential biomarkers of resistance against *C. fumiferana* in *P. glauca* (Mageroy et al., 2015; Méndez‐Espinoza et al., 2018; Parent et al., 2017). Piceol and pungenol also occur in several other species of spruce (*Picea* spp.) and are involved with biotic or abiotic stress responses (Hoque, 1985; Løkke, 1990; Parent, Giguère, Mageroy, Bohlmann, & MacKay, 2018; Strunz, Giguère, & Thomas, 1986). In recent years, genes and enzymes responsible for the accumulation of piceol and pungenol were identified in *P. glauca* foliage using genomic, molecular, and biochemical approaches.

**Figure 1:**
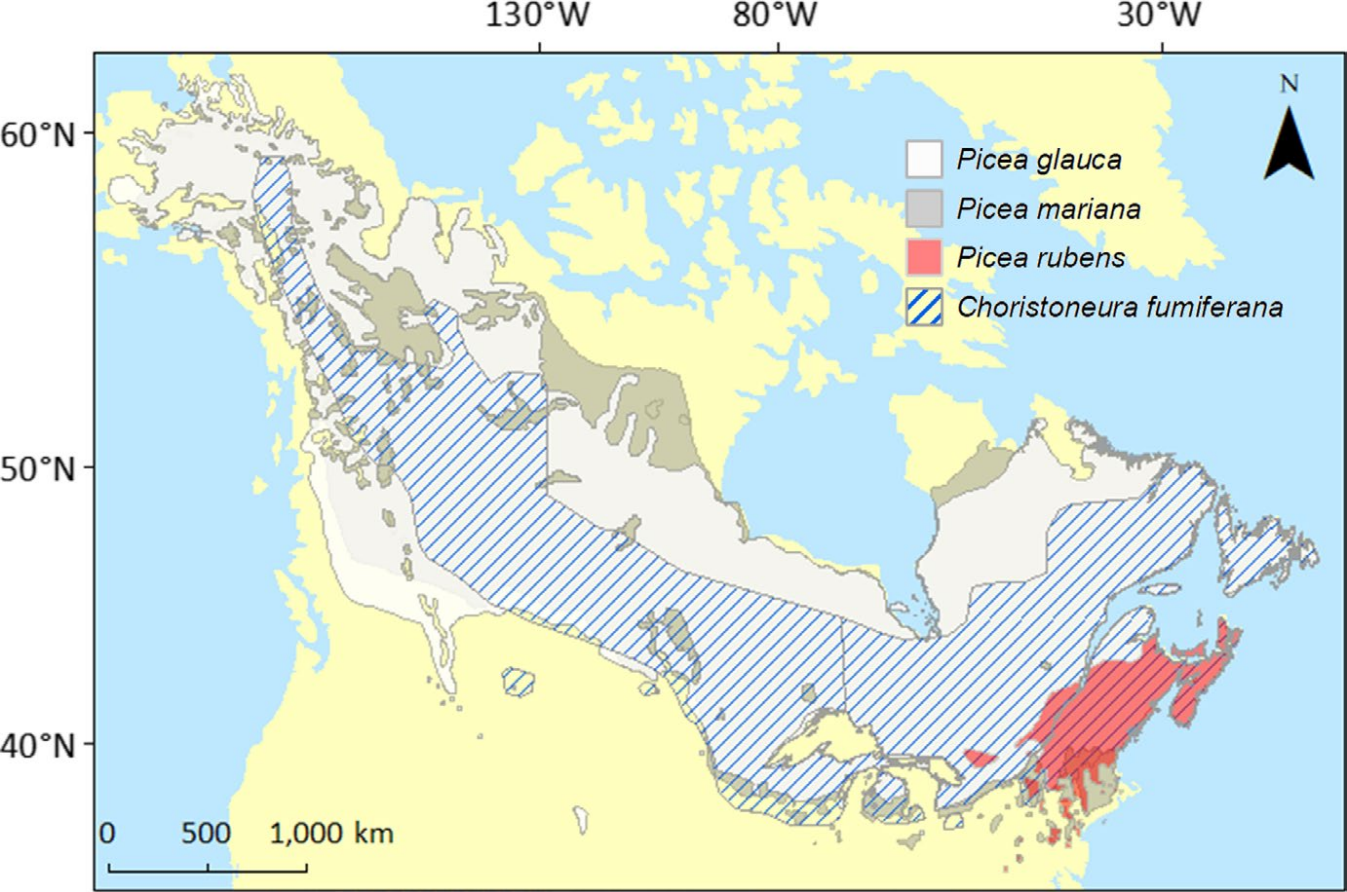
Distribution of North American indigenous spruces (*Picea glauca*, *Picea mariana,* and *Picea rubens*) and spruce budworm (*Choristoneura fumiferana*). Map accessed at https://cfs.nrcan.gc.ca/projects/107 (March 30, 2014) and reproduced with permission from the Natural Resources Canada/Canadian Forest Service

The hydroxyacetophenones defense in *P. glauca* appears to play a role in limiting insect defoliation (Parent et al., 2017), which may impact *C. fumiferana* outbreaks. *Picea glauca* and *C. fumiferana* are native to North America and are distributed from the Canadian Rocky Mountains to the Atlantic (Figure 1). Both species are of large importance to the Canadian forest industry as a major timber species and forest pest, respectively. *Choristoneura fumiferana* has co‐evolved with its primary hosts of spruce (*P. glauca*, *Picea mariana*, *Picea rubens*) and fir (*Abies balsamea*) species for millennia (Simard, Morin, & Potelle, 2002; Figure 1). Epidemic levels of *C. fumiferana* damage covered an area of 850,000 km^2^ in Canada in 1950–93, killing 45%–58% of its hosts in highly affected areas and substantially decreasing wood yields (Gray & MacKinnon, 2006). Defoliation by *C. fumiferana* occurs from early spring when second instar larvae emerges from diapause and mine old foliage. At bud break, the larvae feed on current‐year foliage and undergo four additional larval stadia, the last one being the most damaging, before turning into pupae at mid‐summer. Tree mortality can occur after 3–4 years of severe defoliation.

Prior to the identification of *P. glauca* individuals that resisted attack by *C. fumiferana* (Daoust et al., 2010) and putative resistance compounds (Delvas, Bauce, Labbé, Ollevier, & Bélanger, 2011), there was a lack of evidence to develop an understanding of strong resistance to *C. fumiferana* in any of its host species. The research that we review here has filled crucial knowledge gaps, enabling the development of new hypotheses on the evolution and population dynamics of resistance and the development of a strategy for resistance breeding in *P. glauca*. There are three main hypotheses that have underpinned this work on *P. glauca*: (a) the release of and accumulation of foliar hydroxyacetophenones contribute to resistance against *C. fumiferana* and are primarily constitutive, (b) phenotypic variation in foliar levels hydroxyacetophenones results from quantitative genetic differences and affect fitness, and (c) the biosynthesis of hydroxyacetophenones shows variability developmentally, temporally, and spatially.

Here, we summarize the findings on this recently described defense with information from research on both the tree (Section 2) and the insect (Section 3) components in this plant herbivore interaction. We also present new results including a comparison of the impact of *C. fumiferana* feeding on the transcriptome in resistant (R) and susceptible (S, or nonresistant, N‐R, in previous publications) *P. glauca* (Section 4). Previous work and new observations reported here suggest that the resistance mechanism involves both constitutive and induced components that are influenced by the tree genotype. We discuss possible trajectories of the evolution of this resistance in *P. glauca* and other spruce species native to North America as well as possible applications for resistance breeding a (Section 5) and future research directions (Section 6).

## 2 TREE DEFENSE

### 2.1 Biosynthesis of the defense compounds

Piceol and pungenol play a role in the resistance of *P. glauca* against *C. fumiferana*, while their glucosides appear to be noneffective for resistance (Delvas et al., 2011; Mageroy et al., 2015; Parent et al., 2017). The first evidence supporting this finding came from a study that monitored a plantation of mature *P. glauca* over several years during a *C. fumiferana* outbreak (Bauce & Kumbasli, 2007). The study identified trees as R or S based on low or high levels of defoliation by *C. fumiferana*, respectively (Daoust et al., 2010). Piceol and pungenol accumulated in R trees but not in S trees, in contrast to the glucosides accumulated in both R and S (Delvas et al., 2011; Mageroy et al., 2015). A role of these hydroxyacetophenones in defense was supported by results of bioassays with *C. fumiferana* that showed reduced growth in hydroxyacetophenone fed larvae (Delvas et al., 2011; Parent et al., 2017; and see Section 3). Based on a previously proposed pathway for acetophenone biosynthesis (Negrel & Javelle, 2010; Neish, 1959) and findings from work in *P. glauca,* we outlined a biosynthetic route involving three phases that may explain the hydroxyacetophenone phenotypes observed in *P. glauca* foliage (Figure 1 phases I–III; Mageroy et al., 2015; Mageroy, Jancsik, et al., 2017a; Parent et al., 2018).

Phase I involves the formation of the acetophenone skeleton proposed from work in *Nicotiana tabacum*, and phase II involves the formation of glucosyl conjugates. These two phases are generally active in *P. glauca* foliage with conjugates like picein reaching levels of 10% of the dry mass on average (Méndez‐Espinoza et al., 2018). The existence of these two phases is supported by evidence that an enzyme in spruce is able to form hydroxyacetophenone glucosides (Mageroy, Jancsik, et al., 2017a). A UDP‐sugar‐dependent glycosyltransferase *PgUGT5b* was identified, and in vitro assays showed that it glycosylates pungenol to form pungenin (Mageroy, Jancsik, et al., 2017a). In addition, its spatiotemporal expression profile matches the accumulation of hydroxyacetophenone glucosides in developing foliage (Mageroy, Jancsik, et al., 2017a). These findings validate phase II of the proposed pathway and suggest that at least one other glucosyltransferase is required in order to account for both of the hydroxyacetophenone conjugates found in spruce. Hydroxyacetophenones accumulate as inactive glucosides in spruce and are activated by the cleavage of the glucoside bond.

Phase III involves the release of aglycons by hydrolysis of the glucosides. This process varies among genotypes (Méndez‐Espinoza et al., 2018). We showed that expression levels of a β‐glucosidase gene *Pgβglu‐1* explain variability in aglycon levels (Mageroy et al., 2015; Méndez‐Espinoza et al., 2018; Parent et al., 2017). *Pgβglu‐1* was discovered by transcriptome profiling in field‐grown R and S trees (Mageroy et al., 2015). Its transcripts were the most differentially expressed among 24,000 genes tested (i.e., up to 1,000‐fold higher in R than S) and accumulated preferentially in the foliage. The differences between the R and S trees were consistent in different years and at different time points during the growing season as determined by gene‐specific RT‐qPCR analyses (Mageroy et al., 2015). The recombinant PgβGLU‐1 protein was active on both picein and pungenin, releasing the aglycons piceol and pungenol in vitro and in planta following transgenic overexpression in young spruce trees (Mageroy, Lachance, et al., 2017b; Mageroy et al., 2015). We observed strong phenotypic and genetic correlations between the level of *Pgβglu‐1* expression and both piceol and pungenol levels in *P. glauca* populations, supporting the hypothesis that the PgβGLU‐1 enzyme is important for the accumulation of the two hydroxyacetophenone aglycons (Mageroy et al., 2015; Méndez‐Espinoza et al., 2018; Parent et al., 2017). In S trees, the small increase in *Pgβglu‐1* expression did not lead to an accumulation of piceol and pungenol. This could indicate that a higher threshold of transcripts is required to impact metabolism, with mechanism including to post‐transcriptional control or rapid turnover of the aglycon hydroxyacetophenones in the cell. These hypotheses are supported by the experiment with overexpressing seedlings, where aglycon accumulation only occurred after tissue disruption, but not in planta (Mageroy, Lachance, et al., 2017b).

### 2.2 Constitutive mechanism with limiting steps

Several classes of plant defense compounds, such as cyanogenic glucosides and glucosinolates, are stored as inactive conjugates and only released as bioactive molecules when cells are damaged by herbivory (Mithöfer & Boland, 2012). This induced activation is due to the separation of activating enzymes, glucosidases, and glucosides in intact tissue (Morant et al., 2008). In contrast, the release of hydroxyacetophenone aglycons occurs in intact tissue (Mageroy et al., 2015). The accumulation of bioactive hydroxyacetophenones is constitutive in *P. glauca* individuals with a R phenotype (Mageroy et al., 2015; Parent et al., 2017). In R trees, the aglycons reach 1%–3% of dry weight on average without the presence of insects (Méndez‐Espinoza et al., 2018). Phenotyping for hydroxyacetophenones carried out in nine experimental plantations separated by ca. 650 km (3 and 12 degrees latitude and longitude, respectively) also showed highly variable hydroxyacetophenone levels that were not a result of environmental effect or induction following insect damage (Lamara et al., 2018; Mageroy et al., 2015; Méndez‐Espinoza et al., 2018; Parent et al., 2017). Mechanisms by which trees tolerate piceol and pungenol and control their release remain unknown.

### 2.3 Heritable defense trait

Genetic control of the hydroxyacetophenone defense was studied in *P. glauca* by estimating heritability in field‐grown trees by using sib analysis (Méndez‐Espinoza et al., 2018) and computation from a kinship matrix (Parent et al., 2017). Narrow‐sense heritability (*h* ^2^) estimates for defense traits (i.e., for picein, piceol, pungenol, and *Pgβglu‐1* expression) were moderate to high (0.50–0.60) when analyzing 33 full‐sib families; the broad‐sense heritability (*H* ^2^) was slightly higher (0.55–0.70) when determined in 21 clonal lines (Méndez‐Espinoza et al., 2018). Each of the sib analysis studies used data from juvenile trees (9 years old, Table 1) in two sites in distinct bioclimatic regions (Quebec, Canada). The kinship analysis approach similarly gave moderate to high heritability estimates for defense traits (except for picein which was low *h* ^2^ = 0.27) for a group of 211 unrelated mature individuals in a single common garden genotyped with 4,767 SNP markers (Parent et al., 2017).

**Table 1:** Ontogeny of hydroxyacetophenone biosynthesis in the foliage of *Picea glauca*

Age	D^a^	Material	Picein	Piceol	Pungenol	Reference
1	S	Progeny R and S	Undetected	Undetected	Undetected	Mageroy et al. (2015)
3	J	Progeny R and S	4.1 ± 0.3	Undetected	Undetected	Present study
4	J	Progeny R and S	110.6 ± 13.0	12.8 ± 5.2	15.9 ± 6.5	Present study
6	J	Clonal trials	115.4 ± 3.9	15.4 ± 1.2	6.2 ± 0.6	Méndez‐Espinoza et al. (2018)
9	J	Progeny trials	116.3 ± 4.2	9.6 ± 0.7	17.4 ± 1.2	Méndez‐Espinoza et al. (2018)
14	M	Clonal trials	95.4 ± 3.8	35.2 ± 2.2	12.7 ± 0.8	Méndez‐Espinoza et al. (2018)
15	M	Clonal trial	173.5 ± 21.7	26.2 ± 5.6	11.9 ± 3.0	Parent et al. (2017)
17	M	Clonal trial	44.1 ± 6.6	16.1 ± 5.5	9.6 ± 2.5	Present study^b^
35	M	Progeny trial	71.6 ± 9.6	12.7 ± 2.0	6.4 ± 0.8	Parent et al. (2017)
47	M	Open‐pollinated trial	28.8 ± 11.6	6.6 ± 2.2	8.7 ± 1.4	Mageroy et al. (2015)^c^
50	M	Open‐pollinated trial	25.2 ± 5.7	7.6 ± 1.3	10.1 ± 1.6	Mageroy et al. (2015)^c^

Note

Age and D indicate the tree age in years and the developmental stage, respectively. Trees aged 1, 3, and 4 years are from open‐pollinated seeds from resistant (R) and susceptible (S) mother trees (47–50 years in the table) and were grown in greenhouse. All other trees were field‐grown. The progeny and clonal trials were from controlled breeding from North American provenances, whereas the open‐pollinated trial was from local sources (Drummondville, see Mageroy et al., 2015 for more details). Picein, piceol, and pungenol units are mg/g dry tissue. Means (±*SEM*) are presented.

a S, J, and M indicate seedlings, juvenile trees, and mature trees, respectively.

b Means and *SEM* are for R trees only.

c Values for trees sampled in August.

Our findings to date on hydroxyacetophenone aglycons in spruce consistently show that heritability is moderate to high (levels of 0.50 or more) and explained by strong additive genetic effects. Heritability is specific for the trait of interest, the population, and the environmental conditions, and therefore, it may vary between studies (White, Adams, & Neale, 2007). Research into other defense compounds in forest trees reported a range of heritability levels. For example, in angiosperms such as *Eucalyptus tricarpa*, sideroxylonal is a foliar secondary metabolite that functions as a cross‐resistance mechanism against mammals and insects herbivores and showed a high heritability (*h* ^2^ = 0.60) (Andrew, Wallis, Harwood, Henson, & Foley, 2007). Phenolic glucosides and condensed tannins in *Populus tremuloides* showed high broad‐sense heritability estimates (*H* ^2^ = 0.72 and 0.58, respectively) (Stevens, Waller, & Lindroth, 2007). In conifers such as *Pinus radiata*, heritability estimates for the nonvolatile resin metabolites were much higher (*h* ^2^ = 0.91 ± 0.20) than those for phenolics (*h* ^2^ = 0.18 ± 0.11) (Moreira, Zas, & Sampedro, 2013). Many of the studies regarding the resistance mechanisms in conifers are focused on bark tissues and terpenoid compounds (Celedon & Bohlmann, 2019; Peter, 2018; Raffa et al., 2017; Whitehill & Bohlmann, 2019), and a limited number of studies are dedicated to defensive mechanisms in conifer foliage tissue (Erbilgin & Colgan, 2012; Ralph et al., 2006; Wiggins, Forrister, Endara, Coley, & Kursar, 2016).

### 2.4 Metabolic trade‐offs

Trade‐offs are defined as a negative relationship between traits (Reznick, 1985). These are expected to occur in response to the limitations of external and internal resources, which must be directed to growth, defense, and reproduction of the host plant (Züst & Agrawal, 2017). Phenotypic and genotypic correlations between growth and resistance traits served to assess trade‐offs between the underlying biological processes in *P. glauca* juveniles. Correlations between growth and resistance traits are presented as a first and simple approach to test for trade‐off between growth and defense that excludes possible impact on reproduction (Züst & Agrawal, 2017); therefore, results presented here should be considered as indicative of overall trade‐off.

There were no trade‐offs observed for the accumulation of aglycon hydroxyacetophenones or *Pgβglu‐1* transcripts in *P. glauca*. Progeny or clonal trials showed low (i.e., <0.2) to moderate (i.e., 0.2–0.8) positive phenotypic and genetic correlations (0.14–0.30 and 0.12–0.56, respectively) indicating the absence of trade‐off between growth and defense traits (Méndez‐Espinoza et al., 2018). Another study in mature *P. glauca* considered total tree height, stem diameter at breast height and growth ring width and the accumulation of aglycon hydroxyacetophenones or *Pgβglu‐1* transcripts (Lamara et al., 2018). The correlations were also low (−0.01 to 0.10) supporting the absence of trade‐offs between defense biomarkers with all of the growth traits (Lamara et al., 2018). A PCA analysis also illustrated a neutral relationship between the growth and defense, which is consistent with the findings of the correlation test. These two studies suggest that there are no obvious trade‐offs between hydroxyacetophenone defense and growth in juveniles and mature *P. glauca* under the conditions of our experimental design and analysis.

Picein correlates negatively with growth traits only in clonal trials indicating a possible trade‐off for the production of picein that results from genotypic effects. Picein is accumulated in both R and S trees at similar high concentrations in foliage (Mageroy et al., 2015; Parent et al., 2017). The accumulation of picein may thus limit growth due to the accumulation of conjugated glucose within picein in both R and S trees. The recovery of glucose by deglycolysation may be one of the possible explanation in the positive relation between aglycon acetophenone concentration and growth traits.

### 2.5 Developmental and seasonal variation in hydroxyacetophenones

Plant defense mechanisms may vary during development. For instance, concentrations of constitutive monoterpenes are higher in juveniles than in mature *Pinus banksiana* trees (Erbilgin & Colgan, 2012). Resistance to herbivores is most likely influenced by the probability of herbivory during ontogeny (Boege & Marquis, 2005). It is possible, for instance, that an effective chemical or physical defense only occurs at a stage in plant ontogeny when herbivory is most likely to occur. An example in spruce resistance to herbivory is the spatially and temporally restricted deposition of stone cells that protect resistant *Picea sitchensis* against early instar *Pissodes strobi* weevil larvae (Whitehill, Henderson, Schuetz, et al., 2016a; Whitehill, Henderson, Strong, Jaquish, & Bohlmann, 2016b; Whitehill et al., 2019). A similar case may be present with the production of hydroxyacetophenones, and to support this hypothesis, we summarize new data showing that the biosynthesis of hydroxyacetophenones varies between seedlings, juveniles, and mature *P. glauca* as determined by accumulation levels in current‐year foliage (Table 1). We also summarize the results of a previous study showing that the biosynthesis of the defense compounds varies temporally in the current‐year foliage of *P. glauca* (Parent et al., 2017).

Contrasting patterns in the accumulation of *Pgβglu‐1* transcripts and hydroxyacetophenones are observed during the development of *P. glauca* (Table 1). Transcripts of *Pgβglu‐1* are detected in one‐year old seedlings but do not result in the accumulation of piceol and pungenol, which may be due to a lack of glycosylated hydroxyacetophenone (Mageroy et al., 2015, Table 1). Picein and pungenin (the latter not shown in Table 1) are detected in trees as young as 2 years old, and picein concentrations steeply increase from the age 3 to 4 years (Table 1). Piceol and pungenol were detected only from 4 years of age in greenhouse‐grown saplings and in older trees (Table 1). Glycosylated and aglycon forms of hydroxyacetophenones are detected from the age of 6 years and onwards in all field trials tested (Table 1) and remain present in the current‐year foliage in fifty‐year‐old *P. glauca* trees. The sudden increase in the concentration of piceol and pungenol in four‐year‐old trees may be linked with the increasing probability of damage by *C. fumiferana*. Seedlings of *P. glauca* are less affected than older trees by *C. fumiferana* (Baskerville, 1975); however, the exact age or size at which spruce trees are first attacked by *C. fumiferana* is unknown. Other age‐related parameters such as variations in nutrient quality of the foliage do not explain variation in resistance in *P. glauca* (Mattson, Haack, Lawrence, & Slocum, 1991). Ontogeny variation in this defense mechanism may be due to variation in resource allocation and constraints in architectural development.

There is substantial variation in hydroxyacetophenones and *Pgβglu‐1* expression from bud burst to elongation of year foliage of mature *P. glauca* (Mageroy et al., 2015; Parent et al., 2017). Levels of *Pgβglu‐1* transcripts and aglycon hydroxyacetophenones were low early in the growth season. The *Pgβglu‐1* transcripts began to increase in June in both R and S trees, but peaked and remained two orders of magnitude higher in R trees compared with S trees. The piceol and pungenol concentrations followed similar patterns in R trees but stayed low in S trees (Parent et al., 2017). The foliar concentrations of piceol and pungenol stay high until September and persist to the following spring (at least to May) only in R trees (Parent et al., 2017). Levels in aglycon hydroxyacetophenones reached at the end of the growth season were similar to those of previous‐year foliage (i.e., 2 years old) sampled in May (Parent et al., 2017).

### 2.6 Other genes putatively involved in resistance to *Choristoneura fumiferana* in *Picea glauca*

The genetic basis of variation in hydroxyacetophenones and *Pgβglu‐1* expression levels was investigated by using an association study in a population of 211 individuals from a common garden (Lamara et al., 2018; Parent et al., 2017). When analyzed with a multi locus model, the 4,767 SNP markers explained 43% (nine genes), 27% (eight genes), 20% (three genes), and 23% (seven genes) of the phenotypic variation of piceol, pungenol, picein, and *Pgβglu‐1* transcripts levels, respectively. Most of the 33 genes identified were highly expressed in foliage and were foliage preferential, with only a few that were below the microarray detection threshold (Lamara et al., 2018; Raherison et al., 2012).

Sequence similarity indicated that the associated genes included several carbohydrate metabolism enzymes (i.e., xyloglucan endotransglucosylase/hydrolase 8, sugar transporter protein 7, UDP‐D‐glucuronate 4‐epimerase 1, and UDP‐D‐glucuronate 4‐epimerase 4), a glutamine synthetase which may be linked to phenylpropanoid metabolism, putative regulatory proteins including the SOG1 (suppressor of gamma response 1), a NAC transcriptional factor that regulates DNA damage responses, and genes involved in response to different stimuli and stresses (i.e., ascorbate peroxidase, glutathione S‐transferases, and phenylcoumaran benzylic ether reductase1) (Lamara et al., 2018). A small number of genes may control the resistance biomarkers since estimates of narrow‐sense heritability were high. Wider sampling of the genome will likely be necessary to explain more broadly their heritable variation. Besides, we could not identify any genetic variants within the *Pgβglu‐1* gene that were associated to the R or S phenotypes in multiple sequencing efforts. The level of *Pgβglu‐1* expression is likely to be partly explained by *trans* effects such as variations in upstream regulatory sequences but this remains to be tested (Mageroy et al., 2015).

### 2.7 Hydroxyacetophenones in other conifers

**Figure 2:**
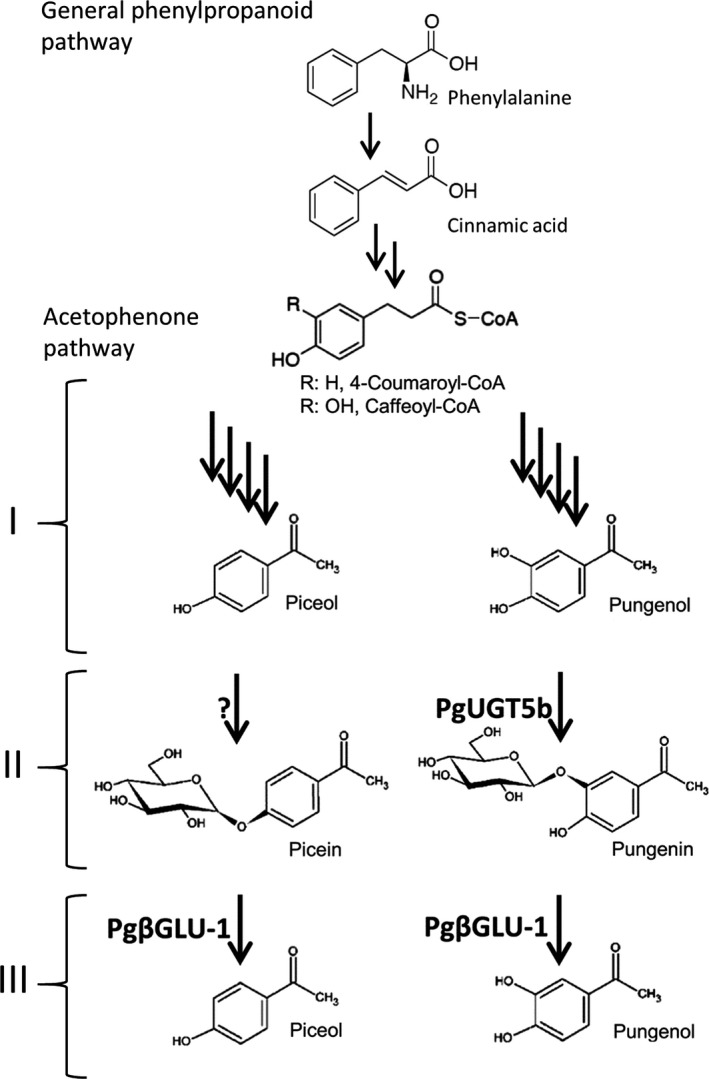
Proposed biosynthesis route for hydroxyacetophenone accumulation patterns in *Picea glauca*. The formation of the acetophenone skeleton was suggested to involve four enzymatic reactions (phase I) (Mageroy, Jancsik, et al., 2017a; Negrel & Javelle, 2010; Neish, 1959) but none of the enzymes are known in plants. Phase II is the formation of glycosyl conjugates such as picein and pungenin found in several spruces. A UDP‐sugar glucosyltransferase PgUGT5b performs this reaction in white spruce, acting specifically on pungenol to form pungenin (Mageroy, Jancsik, et al., 2017a). Phase III is the release of aglycons by hydrolysis of the glucosides, which is attributed to a glucosidase enzyme PgβGLU‐1 in white spruce (Mageroy et al., 2015) and is not functionally demonstrated in other plants

The biosynthesis of hydroxyacetophenones as described for *P. glauca* (Figure 2) appears to be conserved in spruce species (Parent et al., 2018). The role of hydroxyacetophenones in defense may explain the conservation of its biosynthesis across spruce species. Of the spruce species affected by *C. fumiferana* in northeast America (Figure 1), *P. glauca* has the best synchronization between bud burst and elongation and *C. fumiferana* development (Lawrence, Mattson, & Haack, 1997) and has both hydroxyacetophenones defense compounds. In contrast, *P. mariana* and *P. rubens* contain only piceol and no hydroxyacetophenones, respectively, in their current‐year foliage (Parent et al., 2018) and are not synchronized with *C. fumiferana* development. The mismatch between the development of *P. mariana* and *P. rubens*, and *C. fumiferana* has been identified as the main cause for their reduced defoliation. Field rearing experiments have shown increased defoliation when bud phenology of black spruce was artificially matched to that of *C. fumiferana* development (Fuentealba, Pureswaran, Bauce, & Despland, 2017). Another analysis showed that *P. mariana, P. rubens,* and their hybrids are defoliated by *C. fumiferana* with contrasted damage levels (resistance *P. mariana* > hybrids > *P. rubens*, Manley & Fowler, 1969), which matches with their respective hydroxyacetophenone accumulation (Parent et al., 2018). Difference in damage caused by *C. fumiferana* may have a direct impact on species fitness, and thus, the accumulation of hydroxyacetophenones even if only during the last larval stage in new foliage may be vital when the insect and the tree bud development match.

## 3 EFFECTS ON INSECTS

In this section, we describe effects of hydroxyacetophenones on *C. fumiferana* digestion, development, and survival from second instar to adults through different experimental approaches (Delvas et al., 2011; Parent et al., 2017). We also present new results from in vivo experiments that tested the phenogical window of resistance (see Section 3.3). Bioassays used *C. fumiferana* that developed from second instar to the stage of pupae. The experiments mimicked the *C. fumiferana* lifecycle.

### 3.1 Effect of hydroxyacetophenones on *Choristoneura fumiferana* in controlled bioassays

Bioassay results indicated that piceol and pungenol may be active in the defense of *P. glauca* against *C. fumiferana* (Delvas et al., 2011). Second instar larvae were reared in a laboratory incubator on artificial McMorran diet alone (control diet) or supplemented with either piceol, pungenol, or both combined. Bioassays showed that the aglycon hydroxyacetophenones reduced pupal mass (27% female; 24% male), increased development duration (30% female; 22% male), and reduced larval survival (38%) of *C. fumiferana* (Delvas et al., 2011, concentrations 5.4 mg/g for pungenol and 6.7 mg/g for piceol per dry mass). In contrast, a separate study indicated that pungenin had no effect on *C. fumiferana* (Strunz et al., 1986). Delayed development and reduced pupal mass were observed in female and male larvae given the aglycon hydroxyacetophenones‐supplemented diets, but distinct patterns in sensitivity were found in both traits for both sexes. The combined aglycon hydroxyacetophenones reduced the pupal mass in both females and males, whereas piceol alone only reduced female pupal mass (Delvas et al., 2011). In contrast, development was slower in females only in the presence of both of the compounds but in males it was prolonged by piceol alone, pungenol alone, and in combination (Delvas et al., 2011). These effects on growth are likely due to differences in larval performance in the presence of single or combined hydroxyacetophenones. Most notably, the efficiency of conversion of digested and ingested food was increased by the combination of piceol and pungenol, whereas relative growth and consumption rate were reduced compared with those of control diet. Also, larval mortality is negatively affected from the third instar to pupation with increasing concentration of piceol and pungenol in artificial diet (Delvas et al., 2011). These results show that the toxicity of the hydroxyacetophenones seems amplified by additive effects or synergistic interactions of two compounds (Delvas et al., 2011).

The fate of piceol and pungenol after ingestion by the *C. fumiferana* was also studied in a bioassay showing an upregulation of the detoxifying enzyme glutathione S‐transferase and partial conjugation of the hydroxyacetophenones prior to elimination during their ingestion by *C. fumiferana* (Donkor, Mirzahosseini, Bede, Bauce, & Despland, 2019). However, these counter‐measures are not completely effective at mitigating toxic effects of the ingested compounds in bioassays.

The concentrations of hydroxyacetophenones utilized in bioassays may underestimate their effect on *C. fumiferana*. In both studies, the maximum concentrations (i.e., 5.4 mg/g for pungenol and 6.7 mg/g for piceol per dry mass) are lower than their averages in most sampling designs tested (Delvas et al., 2011; Donkor et al., 2019; Table 1). In addition, *C. fumiferana* feeds on previous year's foliage before budburst, which has high levels of hydroxyacetophenones in R trees (Parent et al., 2017). Higher concentrations of piceol and pungenol may have a greater impact on development and survival of *C. fumiferana* as shown in bioassays (Delvas et al., 2011); however, the foliage matrix may limit the absorption of these toxic compounds by *C. fumiferana*.

### 3.2 Effect of R and S adult trees on *Choristoneura fumiferana* in vivo

We compared *C. fumiferana* development and survival on mature *P. glauca* R and S trees using a field insect trial setup during budburst (Parent et al., 2017). Development of female *C. fumiferana* into pupae was slower in R trees, which is consistent with the artificial diet results but no impact was observed on male pupal mass or survival (Delvas et al., 2011; Parent et al., 2017). Differences between bioassays and field trials are often observed and attributed to differences in experimental setup including small sample sizes, insect escapes from the mesh bags, and foliage matrix effect.

The degree of synchrony of spruce trees and *C. fumiferana* phenology also may increase or decrease the effectiveness of the resistance mechanism in *P. glauca*. In 2014, piceol and pungenol concentrations increased only in the resistant trees and started to increase during the sixth instar stage of *C. fumiferana* (Parent et al., 2017). If the insects develop earlier, the resistance mechanism may not be effective and the phenological window of susceptibility to *C. fumiferana* may become wider.

### 3.3 Phenological window of resistance of adult trees on *Choristoneura fumiferana* in vivo

Insect and plant synchrony may define the susceptibility of *P. glauca* to *C. fumiferana* (Lawrence et al., 1997). We examined how they may influence resistance and tested for mismatch (i.e., null or low concentration of piceol and pungenol) and match (i.e., high concentrations of piceol and pungenol) through *C. fumiferana* development by using rearing trials. These experiments were carried out in 2016 on grafted genotypes (or clonal bank in Table 1) established in 1999; the treatments aimed to produce a mismatched (early) and matched (late) synchrony between *P. glauca* and *C. fumiferana* (for details, see methods). We selected eleven R and nine S trees based on hydroxyacetophenone in those genotypes as reported (Mageroy et al., 2015; Parent et al., 2017). Twenty *C. fumiferana* larvae at second instar were placed on two selected branches, enclosed in two fine‐mesh bags, and surveyed for development. Levels of hydroxyacetophenones in the foliage were assayed with liquid chromatography (more details on methods provided in Appendix S1). In the mismatch trial, piceol and pungenol concentrations were low for most of the *C. fumiferana* development and high for the last sampling time point of rearing (June 30). In the matched trial, both compound concentrations were high throughout the development of *C. fumiferana* (Figure S1). Note that the levels of foliar hydroxyacetophenones likely reflected both constitutive and induced defense (see Section 4) since *C. fumiferana* larvae had been installed in all of the trees analyzed here.

There was no effect on any of the insect growth traits or survival in the mismatched resistance trial, whereas lower pupal mass was observed in females in the matched trial (Table 2). In this experiment, the exposure to neither piceol nor pungenol influenced the development and survival of *C. fumiferana* larvae. The impact of hydroxyacetophenones on *C. fumiferana* may seem limited; however, the bioassays most likely underestimate their effect since larvae develop in conditions free of hydroxyacetophenones. Larvae are added at bud break (free of hydroxyacetophenones) to maximize their survival at this critical step of the bioassay; however, they have to feed on previous‐year foliage to develop in natural conditions, which may contain high levels of hydroxyacetophenones. Thus, these results show that duration of exposure to piceol and pungenol influences growth of *C. fumiferana,* indicating that there is a phenological window of resistance. Development and feeding of *C. fumiferana* near the time of bud break could be an adaptation to the *P. glauca* host (Mattson et al., 1991; Nealis, 2012; Zalucki, Clarke, & Malcolm, 2002) when defenses are reduced. We observed differences in survival between mismatched and matched phenological rearing trials, which were consistent with observations that foliage nutritional quality declines quickly with leaf expansion in spruce (Fuentealba & Bauce, 2012; Lawrence et al., 1997; Mattson et al., 1991).

**Table 2:** Effect of phenological stage of *Picea glauca* on defense against *Choristoneura fumiferana*

	Phenological window of defense			
	Mismatched		Matched	
	S	R	S	R
Picein (mg/g)	7.9 ± 2.8	3.9 ± 3.7	31.1 ± 9.6	15.0 ± 8.5
Piceol (mg/g)	0.2 ± 0.2^b^	10.4 ± 3.9^a^	0.1 ± 0.1^b^	34.2 ± 8.3^a^
Pungenol (mg/g)	0.0 ± 0.0^b^	6.5 ± 2.6^a^	0.6 ± 0.4^b^	19.3 ± 4.3^a^
Defoliation (%)	56.8 ± 5.5	68.8 ± 6.0	26.6 ± 7.4	22.0 ± 5.2
Mass female chrysalides (mg)	102.8 ± 4.0	95.3 ± 5.4	70.9 ± 2.9^a^	60.1 ± 2.3^b^
Mass male chrysalides (mg)	69.9 ± 3.2	68.9 ± 4.1	53.7 ± 3.3	48.8 ± 2.6
Development time female (h)	935.6 ± 2.9	931.5 ± 4.8	927.6 ± 13.9	944.7 ± 7.3
Development time male (h)	926.1 ± 9.0	914.9 ± 4.6	914.4 ± 10.1	917.6 ± 5.6
Larval survival (%)	47.1 ± 4.9	44.8 ± 4.0	29.2 ± 3.3	25.0 ± 4.0

Note

Trees of 20 different genotypes classified as resistant (R, *n* = 11) and susceptible (S, *n* = 9) were evaluated in a mismatched (early, May 25 to June 30) and matched (late, June 15–July 19) phenological window of defense. Average values for six and five sampling times per tree are used in either of the mismatched and matched windows, respectively. Superscript letters indicate significant differences (*p* < .05) between the two resistance phenotypes either in the mismatched or in the match window using *t* test. More details on the experimental design are provided in Appendix S1.

## 4 EFFECTS OF TREE AND INSECT INTERACTIONS ON THE TREE TRANSCRIPTOME

Plant possesses both constitutive and inducible defenses. Constitutive defenses provide basal resistance, and inducible defenses afford the conservation of resources in the absence of insect herbivory. We performed a second insect rearing experiment in the field to (a) test whether the hydroxyacetophenone accumulation was inducible and (b) investigate transcriptomic profiles in R and S trees to develop a wider understanding of defense. The small‐scale induction experiment used grafted mature *P. glauca* trees established in 1999. Insects were reared on five genotypes with high (941240, 941290: R) or low (941227, 941239, 941311: S) levels of hydroxyacetophenone aglycons as reported in Parent et al. (2017). We studied two distinct grafts of each genotype (total of ten trees), where one graft received three mesh bags with *C. fumiferana* larvae (treatment) and one graft received an empty mesh bag (control) (total of 20 bags). Bags were placed on June 10, 2015, and current‐year foliage was sampled on June 29, 2015. Levels of hydroxyacetophenones and the transcripts of *Pgβglu‐1* were assayed in each of the 20 samples with liquid chromatography and RT‐PCR, respectively. Transcriptome sequencing (RNA‐Seq) was performed on one *C. fumiferana* treatment sample and one control sample per genotype (total 10 samples). A detailed methodology and more results are presented in Appendix S1.

**Figure 3:**
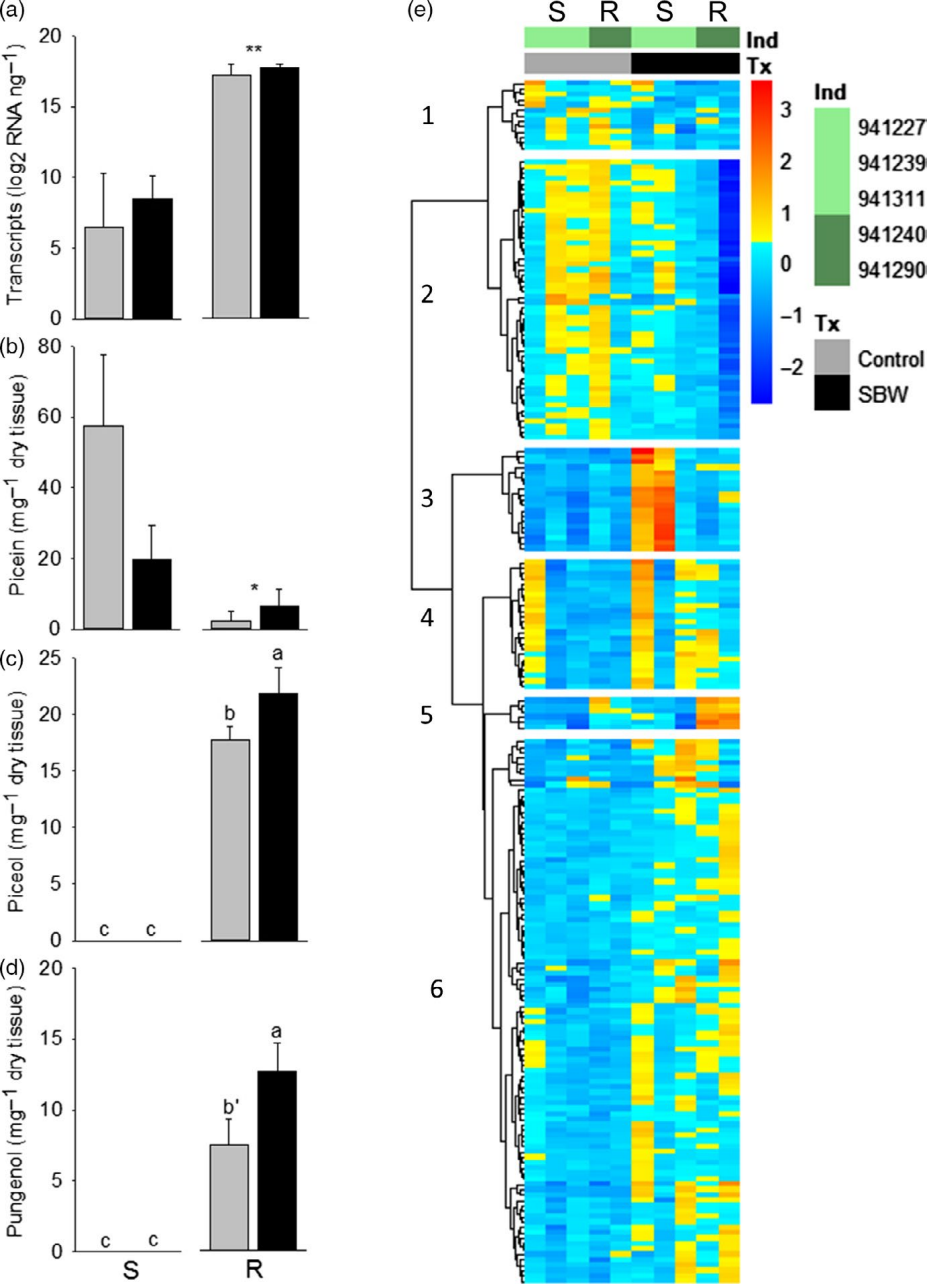
Induction of hydroxyacetophenone defense in the current‐year foliage of mature grafts of *Picea glauca*. Means (±*SEM*) are presented for levels of *Pgβglu‐1* transcripts (a), and hydroxyacetophenones (b)–(d) of control and treated with *Choristoneura fumiferana* trees. We report results of general linear model analyses with treatment and resistance effects and their interactions as sources of variation (using SS4 due to unbalanced data). One or two stars indicate a resistance effect *p* = .06 and *p* = .03, respectively (Table S3). For piceol and pungenol, a significant interaction between treatment and resistance effects was detected (Table S3). Different letters indicate significant differences between the four groups tested for the interaction using a Tukey multiple comparison test (*α* = .05, except for b′ *α* = .08 compared to R treated). (e) Differentially expressed genes in *P. glauca* during herbivory by *C. fumiferana*. The genes presented in this figure are those identified as significantly differentially expressed (Table S4) between the year foliage treated with *C. fumiferana* (SBW for spruce budworm) and its control on a different tree. There are five genotypes with high (941240, 941290: resistant, dark green) or low levels of hydroxyacetophenone aglycons (941227, 941239, 941311: susceptible, pale green). Cells in blue and red are under‐ and overexpressed, respectively (log_2_ scale). The optimal number of clusters for the differentially expressed genes was six based on a Gap PAM statistical method (Figure S5)

The data show that the accumulation of aglycon hydroxyacetophenones is induced only in the R trees with *C. fumiferana* feeding (Figure 3a to d). Piceol and pungenol concentration increased by 23 and 69%, respectively, in the foliage of R trees defoliated by *C. fumiferana* compared with the controls (Figure 3c,d, Table S2). In contrast, levels of *Pgβglu‐1* transcripts were similar between treated and control foliage from R and S trees (Table S3). R trees had lower foliar concentrations of picein than S trees, whereas the levels of picein where similar between R and S trees at other sampling time during that year in trees from the same experimental design (Figure S1) and in previous studies measuring constitutive levels (Parent et al., 2017).

A total of 215 genes were differentially expressed (Figure 3e) between the foliage fed upon by *C. fumiferana* and control trees across all genotypes (Table S4). Note that we compared genotypes that were treated or not with *C. fumiferana* to present the most robust statistical treatment that could detect the effects of the insect with this small sample size. Protein homology with *Arabidopsis thaliana* provided annotations for 138 of the sequences and a total of 124 different TAIR annotations (Table S4). The majority of the differentially expressed genes (DEG; 109) were also detected during damage by *C. fumiferana* on the foliage of *P. sitchensis* using a microarray (Ralph et al., 2006). Response to stress including wounding, biosynthesis of cell walls, and activities related to terpene synthesis are overrepresented in biological and metabolic functions in these annotations (Table S5). Genes linked to the biosynthesis of secondary metabolites and of phenylpropanoids are also overrepresented (Table S5).

A clustering analysis of the differentially expressed genes identified six clusters with distinct patterns of expression (Figure S5). Clusters 1 and 2 contain underexpressed genes in two or more individuals, and clusters 3, 4, 5, and 6 contain overexpressed genes for the *C. fumiferana* treated foliage compared with control (Figure 3e). Genes associated with the biosynthesis of secondary cell wall are in cluster 4, whereas genes associated with secondary metabolites biosynthesis are mostly in clusters 5 and 6 (Table S4). The six genes in cluster 5 are overexpressed in the two *C. fumiferana* treated grafts of the R genotypes and had TAIR annotations either linked to the terpene or phenylpropanoid synthesis (Table 3). *Pgβglu‐1* is in cluster 5 (PG_011735_T.1; Table 3) and has the highest mean level of transcript accumulation across samples (Table S4). Previous work shows that the foliage from R trees contains constitutively greater concentrations of the monoterpenes a‐pinene and myrcene (Daoust et al., 2010). Based on qualitative observations from Figure 3e, some terpene synthase and terpenoid cyclase transcripts are upregulated only in R trees but GO terms for myrcene synthase activity are overrepresented in cluster 6 and expression of the corresponding genes increases in both R and S trees.

**Table 3:** Genes overexpressed in resistant (R) genotypes of *Picea glauca* from the cluster 5 (Figure 3)

Gene	TAIR annotation
PG_007184_T.1	Terpene synthase 02
PG_010124_T.1	Terpene synthase 02
PG_011104_T.1	Ferulic acid 5‐hydroxylase 1
PG_011735_T.1	Beta glucosidase 40
PG_013849_T.1	Terpenoid cyclases/trotein prenyltransferases superfamily protein
PG_014088_T.1	O‐methyltransferase 1

Note

*Pgβglu‐1* gene is PG_011735_T.1.

Results suggest that only R trees respond to *C. fumiferana* attack with the increased biosynthesis of hydroxyacetophenones. Genes from cluster 5 involved in terpenes and other phenolic compounds synthesis seemed upregulated only in R trees; however, larger sample sizes and more time points testing for difference in transcript accumulation between R and S trees are required for such conclusion. Our results also show that the same defense compounds may be constitutive and induced in a given plant species. The classification of defense mechanisms as constitutive or induced is not supported when the defense mechanisms are fully characterized at the molecular level and is complicated by the fact that the same compounds may be involved in both types of defenses in a given plant species (Gatehouse, 2002). Our results show that the individuals identified as producing the hydroxyacetophenones constitutively also produced them at higher levels after induction. This observation may not be specific to *P. glauca,* and a broader conifer species survey may show similar patterns for defense (Parent et al., 2018).

## 5 EVOLUTION AND ARTIFICIAL SELECTION OF HYDROXYACETOPHENONE DEFENSE

### 5.1 Natural evolution

**Figure 4:**
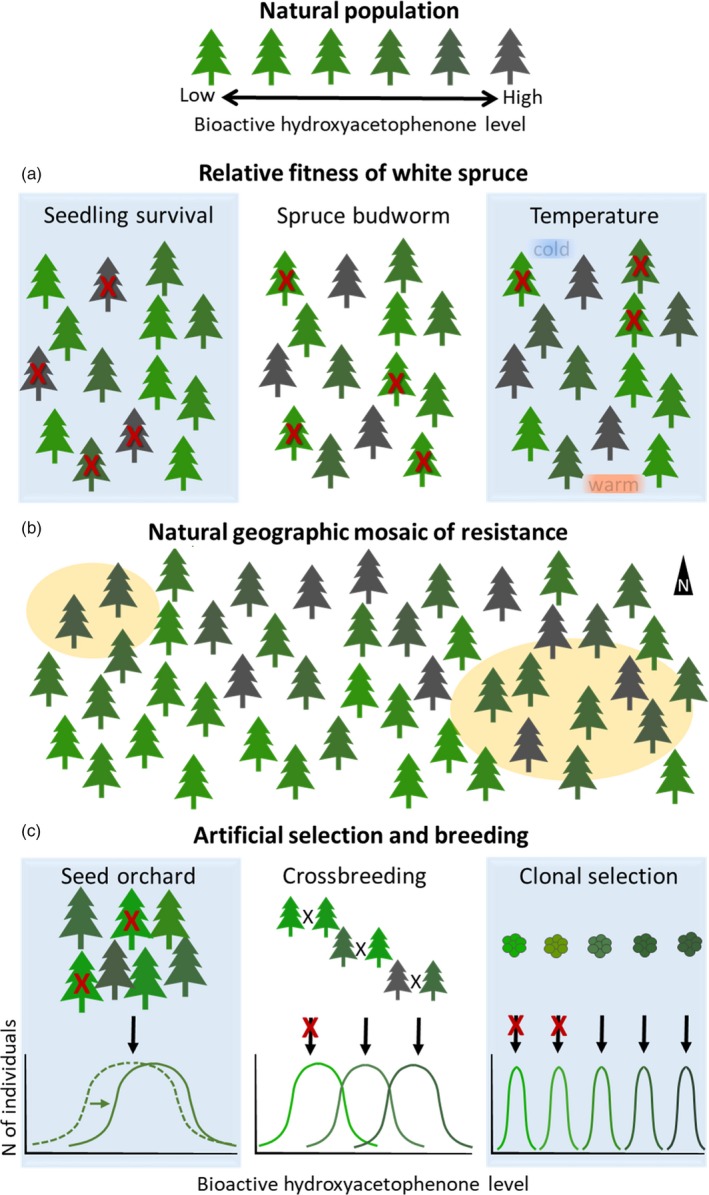
Natural and artificial variation in the frequency of resistant (R) and susceptible (S) in *Picea glauca*. (a) Diagram of relative fitness of trees with high and low content in hydroxyacetophenones. *Left*: Survival of seedling life stage. *Center*: Survival of mature trees under *Choristoneura fumiferana* damage. *Right*: Survival of trees in a temperature gradient. (b) Diagram of the distribution of R and S mature trees that were affected or not by *C. fumiferana* (yellow circles). Information from relative fitness presented in (a) is combined in this diagram. (c) Opportunities for improving *C. fumiferana* resistance in planted *P. glauca* from naturally occurring diversity in *C. fumiferana* resistance biomarkers. Examples of practical genetic selection opportunities and breeding outputs are shown with the potential to increases bioactive hydroxyacetophenone levels. *Left*: Short‐term effects are expected from selective removal of low hydroxyacetophenone genotypes in production seed orchards, resulting in an overall improvement of seed stocks. *Center*: Selection of high hydroxyacetophenone genotypes for use in cross‐pollinations as part of advanced breeding (longer‐term effect) and deployment of full‐sib families (shorter‐term effects). *Right*: Selection of high hydroxyacetophenone clonal materials and deployment of multiclonal varieties, with potential of short‐term effects when selecting somatic embryogenic lines that are in cryogenic storage

This section summarizes the effects of aglycon hydroxyacetophenones on the fitness of *P. glauca* during its life cycle and in its distribution. Negative and positive effects of hydroxyacetophenones were observed on the fitness in Pinaceae species (Figure 4a). We integrate all of the evidence‐based information on relative fitness in a synthesis diagram to provide an integrated hypothesis of the evolution of this defense mechanism in *P. glauca* in eastern Canada (Figure 4b).

Aglycon hydroxyacetophenones may have an impact in the fitness of *P. glauca* in natural populations for survival of offspring exposed to epidemics of *C. fumiferana* and cold ambient temperatures (Parent et al., 2017). Early in development, allelopathy due to pungenol from foliage leachate decreased seed germination in *Picea schrenkiana* (Ruan et al., 2011) indicating a negative effect of exogenous exposure on fitness (Figure 4a). At low latitude, ambient temperature is usually higher and trees with high concentration of aglycon hydroxyacetophenones may have lower fitness due to lower offspring survival (Figure 4b). At maturity, the fitness of *P. glauca* may improve by endogenous accumulation to high concentrations of piceol and pungenol, which limit the herbivory and slow the development of *C. fumiferana* (Delvas et al., 2011; Parent et al., 2017; this study, Figure 4a). Trees with high concentrations of hydroxyacetophenone were also more abundant in areas historically affected by *C. fumiferana* (Parent et al., 2017), suggesting a higher survival or fitness of R trees in these areas (Figure 4b). Concentrations of hydroxyacetophenones are also reported to increase with latitude in *P. glauca* and in *Picea abies*, the latter not being affected by *C. fumiferana* in its natural distribution (Parent et al., 2017; Rummukainen, Julkunen‐Tiitto, Räisänen, & Lehto, 2007). Piceol and pungenol may play a role in increasing survival at high latitudes (Figure 4a). Thus, R trees may be more frequent at northern latitude (Figure 4b).

The synthesis diagram (Figure 4b) reflects the geographic variation in the presence of R trees across eastern distribution of *P. glauca* (Parent et al., 2017). The strong latitudinal temperature gradient and the recurrent selection pressure over the last hundreds or thousand years by *C. fumiferana* are likely caused by the latitudinal changes in the proportion of R trees in the natural population (Parent et al., 2017; Simard et al., 2002). It was hypothesized that variation in the relative fitness of *P. glauca* during the development and across the landscape may avoid the fixation of one phenotype within the tree population and, thus, the fixation of a counter‐response to the tree defense (Parent et al., 2017). Insects can develop counter‐adaptations to plant defensive traits that are heritable and enable the herbivores to withstand plant defense. For instance, *C. fumiferana* can respond to plant hydroxyacetophenones by upregulating a GST to partially eliminate the compounds (Donkor et al., 2019). This counter‐adaptation could challenge the survival of the plant. Counter‐adaptations in insects are often observed in insect pest management programmes for agriculture when only the R phenotype is present (Richard Karban & Agrawal, 2002). In natural forest, an insect with a counter‐adaptation to a toxin could be highly devastating as it can migrate but given the high variability of hydroxyacetophenones in R phenotypes across the eastern distribution of *P. glauca*, this is unlikely to happen. However, in plantations, the likelihood of counter‐adaptation may increase which suggests that strategy of deployment of R trees in plantation should consider mixing resistant with S trees to dilute counter‐adaptation development.

A northward shift of the *C. fumiferana* distribution has been observed since the beginning of the 20th century moving from the *P. glauca* range into the *P. mariana* range (Navarro, Morin, Bergeron, & Montoro Girona, 2018). Geographic variation in resistance to *C. fumiferana* in *P. glauca* allows us to predict that *P. glauca* at the Nordic distribution may survive better to a new or more intensive threat from *C. fumiferana* due to a higher content of foliar hydroxyacetophenones, whereas *P. mariana* may be highly affected by *C. fumiferana* since it contains only one of the two hydroxyacetophenones.* Picea mariana* could fend off *C. fumiferana* attack with other biochemical defenses, but recent studies have shown that it is a good‐quality host when bud phenology matches with *C. fumiferana* development (Fuentealba et al., 2017). A further survey for geographic patterns in biomarker resistance for *P. mariana* may help to predict the impact of this insect on populations.

### 5.2 Artificial selection and resistance breeding

The research summarized here opens the doors to *C. fumiferana* resistance breeding in *P. glauca*, an important forest resource in North America (Méndez‐Espinoza et al., 2018). Breeding in forest trees uses naturally occurring phenotypic variability to develop germplasm for commercial planting by the use of recurrent genetic selection, cross‐pollinations, and propagation through seed production or vegetative multiplication (White et al., 2007). The effort required to change phenotypes through recurrent selection is inversely proportional to the level of trait heritability (Geber & Griffen, 2003). Here, Figure 4c illustrates diverse opportunities for *C. fumiferana* resistance breeding aiming to increase constitutive hydroxyacetophenone levels in *P. glauca* including application in the short and long term. Breeding objectives may include selection for hydroxyacetophenone levels as discussed here as well as defensive terpene metabolites (Daoust et al., 2010). Breeding work may also benefit from the development of molecular markers for genomic selection, and knowledge of the inducible defenses against *C. fumiferana* attack as indicated by RNA‐Seq results is reported here (Lamara et al., 2018). In addition, the *C. fumiferana* resistance biomarkers have been detected in other species to variable extents, specifically suggesting a potential for breeding in *P. mariana* and *P. abies* (Parent et al., 2018).

## 6 FUTURE RESEARCH DIRECTIONS

Considerable progress has been made in understanding the role of hydroxyacetophenones in *P. glauca* resistance to *C. fumiferana*. Recent research has provided insights into both the molecular and genetic underpinnings of this chemical defense. The robustness of hydroxyacetophenone‐based resistance in the natural population facilitated its elucidation. The identification and the characterization of the biosynthetic genes behind this defense are rare examples of functional genetics in a nonmodel tree species. While we have identified two key genes in the biosynthesis of hydroxyacetophenones, we still do not know how expression of these genes is regulated. Many layers of regulation, such as *cis*‐, *trans*‐, epigenetic, and post‐transcriptional regulation, may be involved in the developmental, seasonal, and genetic differential expression of *Pgβglu‐1*. Additionally, we do not have any knowledge about the role of cellular compartmentalization in accumulation and/or release of hydroxyacetophenone glucosides and aglycons. Compartmentalization could play a big part in the ability of PgBLU1 to access its substrate and the metabolic flux through the hydroxyacetophenone biosynthesis pathway. A better understanding of both gene regulation and cellular compartmentalization may be important for the implementation of PgBGLU1 as a marker in breeding programs.

Through our work on *P. glauca* hydroxyacetophenones, we have also gained some insight into the evolution of acetophenones in *Picea* spp. and across land plants. In contrast, the co‐evolution of *P. glauca* and *C. fumiferana* is still poorly understood. *Picea glauca* and *C. fumiferana* have been cohabitating for thousands of year. However, the northern shift of *C. fumiferana* outbreaks indicates that defoliator may be moving into naïve populations. Reared *C. fumiferana* larvae do seem to have some hydroxyacetophenone detoxification mechanisms although not robust enough to overcome hydroxyacetophenone‐based resistance. In a natural *C. fumiferana* population, more effective detoxification mechanisms that are not apparent in these genetically limited colonies may have evolved. Monogenic resistance is not thought to be a durable control of disease in crop systems as targeted pest rapidly breaks this resistance. However, *P. glauca* resistance to *C. fumiferana* in a natural environment is probably more complex than just the ability to cleave hydroxyacetophenone glucosides, as indicated by transcriptome analysis present here. Thus, a better understanding of the evolutionary dynamics between resistant *P. glauca* populations and *C. fumiferana* populations will be vital for effective utilization of hydroxyacetophenone defenses in breeding programs.

## CONFLICT OF INTEREST

None declared.

## Data Availability

Data for this study are available at https://doi.org/10.5061/dryad.n02v6wwsb.(Parent et al., 2019)
