# Supplementary material for: Hydroxyacetophenone defenses in white spruce against spruce budworm
Source: Evol Appl. 2019 Dec 20;13(1):62–75. doi: 10.1111/eva.12885 (PMC6935585; doi:10.1111/eva.12885)
Supplement: Supplementary file 1 [file EVA-13-62-s001.docx]

## Appendix S1

### Table S1

Characteristics of *Picea glauca* used for ontogenic variation in defense in foliage. R: resistant, S: susceptible.

| Age | Type of plant material | Location |
| --- | --- | --- |
| 1 | Progeny from R and S mother | Greenhouse |
| 3 | Progeny from R and S mother | Greenhouse |
| 4 | Progeny from R and S mother | Greenhouse |
| 6 | Clonal trials | Grandes Piles (46°41’N, 72°43’W),  Saint-Modeste (47°50’N, 69°30’W) |
| 9 | Progeny trials | Grandes Piles (46°41’N, 72°43’W),  Normandin (48°50’N, 72°32’W) |
| 14 | Clonal trials | Black Brook (47°19’N, 67°39’W),  Deersdale (46°30’N, 67°4’W),  Parkindale (45°52’N, 65°4’W) |
| 15 | Clonal trial | Valcartier (46°56’N, 71°29’W) |
| 17 | Clonal trial | Valcartier (46°56’N, 71°29’W) |
| 35 | Progeny trial | Mastigouche (46°38’N, 73°13’W) |
| 47 | Local open-pollinated seeds | Drummondville (45°93’N,72°52’W) |
| 50 | Local open-pollinated seeds | Drummondville (45°93’N,72°52’W) |

### Table S2

Induction of the defense mechanism in mature grafts of *Picea glauca*. Hydroxyacetophenones and transcripts were assayed using liquid chromatography and RT-PCR, respectively (more details in methods of SuppInfo.). C: control, T: *Choristoneura fumiferana* treatment, N: number bags analyzed per tree.

| Genotype | Resistance | Treatment | N | Transcripts | Picein | Piceol | | Pungenol | |  |
| --- | --- | --- | --- | --- | --- | --- | --- | --- | --- | --- |
|  |  |  |  | (log_2_ ng^-1^ RNA) | (mg g^-1^) | (mg g^-1^) | | (mg g^-1^) | |  |
| 941227 | S | C | 1 | 13.3 | 17.6 | 0.0 | | 0.0 | |  |
| 941239 | S | C | 1 | 0.0 | 78.3 | 0.0 | | 0.0 | |  |
| 941311 | S | C | 1 | 6.3 | 76.5 | 0.0 | | 0.0 | |  |
| 941240 | R | C | 1 | 17.9 | 0.0 | 16.6 | | 5.7 | |  |
| 941290 | R | C | 1 | 16.4 | 4.9 | 18.9 | | 9.4 | |  |
|  |  |  |  |  |  | |  | |  | |
| 941227 | S | T | 3 | 13.3 ±0.4 | 0.0 ± 0.0 | 0.0 ± 0.0 | | 0.0 ± 0.0 | |  |
| 941239 | S | T | 3 | 3.5 ± 1.7 | 40.6 ± 19.5 | 0.0 ± 0.0 | | 0.0 ± 0.0 | |  |
| 941311 | S | T | 3 | 8.6 ± 1.6 | 18.6 ± 18.0 | 0.0 ± 0.0 | | 0.0 ± 0.0 | |  |
| 941240 | R | T | 3 | 18.6 ± 0.1 | 3.8 ± 3.9 | 23.5 ± 4.9 | | 10.4 ± 3.1 | |  |
| 941290 | R | T | 3 | 17.3 ± 0.4 | 9.5 ± 9.4 | 20.3 ± 0.9 | | 15.0 ± 2.0 | |  |

### Table S3

Results of the statistical analysis of the induction of the defense mechanism in mature grafts of *Picea glauca*. We used a GLM with treatment and control trees (Tx) on five different genotypes either resistant or susceptible to *Choristoneura fumiferana* (Res). DF: degree of freedom, SS: sum of square. See Fig. 3 for more details.

| Source | DF | SS | Mean square | F value | P value |
| --- | --- | --- | --- | --- | --- |
| *Pg βglu-1 transcripts* |  |  |  |  |  |
| Tx | 1 | 3.7 | 3.7 | 0.2 | 0.70 |
| Res | 1 | 236.4 | 236.4 | 10.3 | 0.02 |
| Tx*Res | 1 | 1.2 | 1.2 | 0.1 | 0.83 |
|  |  |  |  |  |  |
| *Picein* |  |  |  |  |  |
| Tx | 1 | 672.7 | 672.7 | 1.3 | 0.31 |
| Res | 1 | 2778.5 | 2778.5 | 5.2 | 0.06 |
| Tx*Res | 1 | 1057.6 | 1057.6 | 2.0 | 0.21 |
|  |  |  |  |  |  |
| *Piceol* |  |  |  |  |  |
| Tx | 1 | 10.3 | 10.3 | 8.0 | 0.03 |
| Res | 1 | 943.3 | 943.3 | 728.9 | <.0001 |
| Tx*Res | 1 | 10.3 | 10.3 | 8.0 | 0.03 |
|  |  |  |  |  |  |
| *Pungenol* |  |  |  |  |  |
| Tx | 1 | 15.9 | 15.9 | 5.5 | 0.06 |
| Res | 1 | 246.0 | 246.0 | 84.7 | <.0001 |
| Tx*Res | 1 | 15.9 | 15.9 | 5.5 | 0.05 |

### Table S4

Genes differentially expressed in the year foliage of *Picea glauca* between control and treated with *Choristoneura fumiferana* trees with the same genotype. The genes were identified using DEseq2 v1.20.0 with the likelihood ratio test method considering genotype as a nested factor of treatment. The base mean represents the mean expression (counts) of the gene over all libraries. The fold change indicates the log_2_ change in expression between control and treated with *C. fumiferana* trees. The cluster numbers are from Fig. S5, respecting the order from top to bottom in the phylogeny, based on the Gap partitioning around medoids statistical method (see methods for more details). N/A indicates that no function was associated to the white spruce gene.

| Gene | Base Mean | Fold Change | P value | Cluster | TAIR gene | TAIR annotation |
| --- | --- | --- | --- | --- | --- | --- |
| PG_028428_T.1 | 300 | -2.9 | 0.009 | 2 | N/A | N/A |
| PG_029690_T.1 | 173 | -2.1 | 0.020 | 2 | N/A | N/A |
| PG_009261_T.1 | 127 | -1.7 | 0.014 | 2 | ATCG01280 | Chloroplast Ycf2;ATPase, AAA type, core |
| PG_028587_T.1 | 40 | -1.7 | 0.003 | 2 | N/A | N/A |
| PG_021163_T.1 | 34 | -1.6 | 0.018 | 2 | N/A | N/A |
| PG_029438_T.1 | 61 | -1.5 | 0.037 | 2 | N/A | N/A |
| PG_009930_T.1 | 37 | -1.5 | 0.032 | 2 | N/A | N/A |
| PG_026133_T.1 | 37 | -1.5 | 0.031 | 2 | N/A | N/A |
| PG_017458_T.1 | 38 | -1.5 | 0.006 | 2 | N/A | N/A |
| PG_035669_T.1 | 45 | -1.5 | 0.044 | 2 | N/A | N/A |
| PG_027200_T.1 | 36 | -1.5 | 0.023 | 2 | N/A | N/A |
| PG_035996_T.1 | 34 | -1.5 | 0.026 | 2 | N/A | N/A |
| PG_034491_T.1 | 43 | -1.5 | 0.027 | 2 | N/A | N/A |
| PG_028603_T.1 | 25 | -1.4 | 0.024 | 2 | N/A | N/A |
| PG_025787_T.1 | 118 | -1.4 | 0.022 | 2 | N/A | N/A |
| PG_027949_T.1 | 45 | -1.4 | 0.005 | 2 | N/A | N/A |
| PG_021715_T.1 | 134 | -1.4 | 0.044 | 2 | N/A | N/A |
| PG_030262_T.1 | 56 | -1.4 | 0.037 | 2 | N/A | N/A |
| PG_020878_T.1 | 63 | -1.3 | 0.026 | 2 | N/A | N/A |
| PG_024502_T.1 | 54 | -1.3 | 0.032 | 2 | N/A | N/A |
| PG_033470_T.1 | 114 | -1.3 | 0.026 | 2 | N/A | N/A |
| PG_029606_T.1 | 94 | -1.3 | 0.049 | 2 | N/A | N/A |
| PG_022594_T.1 | 31 | -1.3 | 0.032 | 2 | N/A | N/A |
| PG_025517_T.1 | 30 | -1.3 | 0.018 | 2 | N/A | N/A |
| PG_026275_T.1 | 47 | -1.3 | 0.034 | 2 | N/A | N/A |
| PG_030667_T.1 | 86 | -1.2 | 0.045 | 2 | N/A | N/A |
| PG_006253_T.1 | 62 | -1.2 | 0.040 | 2 | N/A | N/A |
| PG_016990_T.1 | 52 | -1.2 | 0.028 | 2 | N/A | N/A |
| PG_024690_T.1 | 36 | -1.2 | 0.025 | 2 | N/A | N/A |
| PG_026164_T.1 | 73 | -1.2 | 0.049 | 2 | AT4G23160 | cysteine-rich RLK (RECEPTOR-like protein kinase) 8 |
| PG_022284_T.1 | 62 | -1.2 | 0.014 | 2 | N/A | N/A |
| PG_024527_T.1 | 29 | -1.2 | 0.014 | 1 | AT1G69550 | disease resistance protein (TIR-NBS-LRR class) |
| PG_027111_T.1 | 42 | -1.2 | 0.048 | 2 | N/A | N/A |
| PG_029734_T.1 | 47 | -1.2 | 0.040 | 2 | N/A | N/A |
| PG_027912_T.1 | 193 | -1.1 | 0.049 | 2 | N/A | N/A |
| PG_020868_T.1 | 54 | -1.1 | 0.046 | 2 | N/A | N/A |
| PG_002570_T.1 | 96 | -1.1 | 0.021 | 2 | N/A | N/A |
| PG_025308_T.1 | 93 | -1.1 | 0.043 | 2 | N/A | N/A |
| PG_018710_T.1 | 28 | -1.1 | 0.003 | 2 | N/A | N/A |
| PG_028348_T.1 | 66 | -1.1 | 0.041 | 2 | N/A | N/A |
| PG_031459_T.1 | 50 | -1.0 | 0.044 | 2 | AT1G26560 | beta glucosidase 40 |
| PG_006481_T.1 | 31 | -1.0 | 0.008 | 1 | AT2G34930 | disease resistance family protein / LRR family protein |
| PG_019478_T.1 | 26 | -1.0 | 0.016 | 2 | AT3G42640 | H(+)-ATPase 8 |
| PG_009224_T.1 | 87 | -1.0 | 0.002 | 1 | N/A | N/A |
| PG_011899_T.1 | 24 | -1.0 | 0.038 | 2 | AT1G50030 | target of rapamycin |
| PG_024550_T.1 | 22 | -1.0 | 0.037 | 1 | N/A | N/A |
| PG_007631_T.1 | 34 | -0.9 | 0.017 | 1 | AT2G47000 | ATP binding cassette subfamily B4 |
| PG_029313_T.1 | 23 | -0.9 | 0.014 | 1 | N/A | N/A |
| PG_035590_T.1 | 59 | -0.9 | 0.002 | 1 | N/A | N/A |
| PG_022772_T.1 | 63 | -0.9 | 0.019 | 2 | N/A | N/A |
| PG_032580_T.1 | 77 | -0.9 | 0.048 | 2 | AT4G23160 | cysteine-rich RLK (RECEPTOR-like protein kinase) 8 |
| PG_033175_T.1 | 52 | -0.9 | 0.044 | 2 | AT3G22220 | hAT transposon superfamily |
| PG_018457_T.1 | 53 | -0.9 | 0.042 | 2 | N/A | N/A |
| PG_024872_T.1 | 24 | -0.9 | 0.033 | 2 | N/A | N/A |
| PG_015227_T.1 | 38 | -0.9 | 0.018 | 1 | N/A | N/A |
| PG_017966_T.1 | 55 | -0.8 | 0.048 | 2 | N/A | N/A |
| PG_011875_T.1 | 44 | -0.8 | 0.049 | 1 | AT5G17420 | Cellulose synthase family protein |
| PG_022825_T.1 | 29 | -0.8 | 0.048 | 2 | N/A | N/A |
| PG_007495_T.1 | 56 | -0.8 | 0.014 | 1 | N/A | N/A |
| PG_002445_T.1 | 80 | -0.8 | 0.038 | 2 | N/A | N/A |
| PG_025645_T.1 | 48 | -0.7 | 0.045 | 2 | AT1G78340 | glutathione S-transferase TAU 22 |
| PG_028223_T.1 | 80 | -0.7 | 0.031 | 2 | N/A | N/A |
| PG_023575_T.1 | 96 | -0.7 | 0.045 | 1 | AT3G47580 | Leucine-rich repeat protein kinase family protein |
| PG_027547_T.1 | 93 | -0.7 | 0.014 | 1 | AT1G56130 | Leucine-rich repeat transmembrane protein kinase |
| PG_022990_T.1 | 111 | -0.5 | 0.047 | 1 | AT1G56145 | Leucine-rich repeat transmembrane protein kinase |
| PG_013465_T.1 | 50 | 0.6 | 0.043 | 6 | AT5G42020 | Heat shock protein 70 (Hsp 70) family protein |
| PG_016139_T.1 | 51 | 0.6 | 0.033 | 6 | AT5G45160 | Root hair defective 3 GTP-binding protein (RHD3) |
| PG_008081_T.1 | 53 | 0.6 | 0.048 | 6 | AT3G03940 | Protein kinase family protein |
| PG_016616_T.1 | 69 | 0.6 | 0.030 | 6 | AT5G22060 | DNAJ homologue 2 |
| PG_014067_T.1 | 64 | 0.6 | 0.039 | 6 | AT3G16630 | P-loop containing nucleoside triphosphate hydrolases superfamily protein |
| PG_013751_T.1 | 41 | 0.6 | 0.043 | 6 | AT1G79530 | glyceraldehyde-3-phosphate dehydrogenase of plastid 1 |
| PG_007627_T.1 | 107 | 0.6 | 0.032 | 6 | AT5G15450 | casein lytic proteinase B3 |
| PG_014329_T.1 | 48 | 0.6 | 0.038 | 6 | AT3G13224 | RNA-binding (RRM/RBD/RNP motifs) family protein |
| PG_006618_T.1 | 35 | 0.6 | 0.031 | 6 | AT4G31180 | Class II aminoacyl-tRNA and biotin synthetases superfamily protein |
| PG_003379_T.1 | 43 | 0.6 | 0.038 | 6 | AT4G30600 | signal recognition particle receptor alpha subunit family protein |
| PG_003780_T.1 | 45 | 0.7 | 0.049 | 6 | AT1G59900 | pyruvate dehydrogenase complex E1 alpha subunit |
| PG_004651_T.1 | 51 | 0.7 | 0.017 | 6 | AT2G28310 | Protein of unknown function (DUF707) |
| PG_009187_T.1 | 85 | 0.7 | 0.042 | 6 | AT1G80160 | Lactoylglutathione lyase / glyoxalase I family protein |
| PG_010950_T.1 | 31 | 0.7 | 0.050 | 6 | AT1G23740 | Oxidoreductase, zinc-binding dehydrogenase family protein |
| PG_004909_T.1 | 40 | 0.7 | 0.048 | 6 | AT5G13420 | Aldolase-type TIM barrel family protein |
| PG_005493_T.1 | 58 | 0.7 | 0.017 | 6 | AT3G02360 | 6-phosphogluconate dehydrogenase family protein |
| PG_008300_T.1 | 47 | 0.7 | 0.033 | 6 | AT5G56680 | Class II aminoacyl-tRNA and biotin synthetases superfamily protein |
| PG_005766_T.1 | 80 | 0.7 | 0.041 | 6 | AT4G33070 | Thiamine pyrophosphate dependent pyruvate decarboxylase family protein |
| PG_005986_T.1 | 39 | 0.7 | 0.037 | 6 | AT3G25230 | rotamase FKBP 1 |
| PG_010866_T.1 | 73 | 0.7 | 0.024 | 4 | AT2G38120 | Transmembrane amino acid transporter family protein |
| PG_026582_T.1 | 44 | 0.7 | 0.050 | 6 | AT1G77330 | 2-oxoglutarate (2OG) and Fe(II)-dependent oxygenase superfamily protein |
| PG_002877_T.1 | 43 | 0.7 | 0.029 | 6 | AT1G79750 | NADP-malic enzyme 4 |
| PG_006612_T.1 | 51 | 0.7 | 0.034 | 6 | AT2G02010 | glutamate decarboxylase 4 |
| PG_012064_T.1 | 38 | 0.7 | 0.031 | 6 | AT1G54100 | aldehyde dehydrogenase 7B4 |
| PG_005505_T.1 | 53 | 0.7 | 0.016 | 4 | AT3G21190 | O-fucosyltransferase family protein |
| PG_015817_T.1 | 57 | 0.7 | 0.014 | 6 | AT2G27050 | ETHYLENE-INSENSITIVE3-like 1 |
| PG_005735_T.1 | 111 | 0.7 | 0.032 | 6 | AT5G60600 | 4-hydroxy-3-methylbut-2-enyl diphosphate synthase |
| PG_016336_T.1 | 28 | 0.7 | 0.045 | 6 | AT5G53750 | CBS domain-containing protein |
| PG_001784_T.1 | 68 | 0.7 | 0.015 | 6 | AT4G02280 | sucrose synthase 3 |
| PG_029358_T.1 | 123 | 0.7 | 0.026 | 6 | AT1G55020 | lipoxygenase 1 |
| PG_008669_T.1 | 68 | 0.7 | 0.006 | 6 | AT5G42080 | dynamin-like protein |
| PG_005578_T.1 | 59 | 0.7 | 0.018 | 4 | AT3G10320 | Glycosyltransferase family 61 protein |
| PG_014176_T.1 | 35 | 0.7 | 0.031 | 6 | AT3G51850 | calcium-dependent protein kinase 13 |
| PG_013767_T.1 | 78 | 0.7 | 0.040 | 4 | AT4G18780 | cellulose synthase family protein |
| PG_001877_T.1 | 124 | 0.7 | 0.022 | 6 | AT2G39770 | Glucose-1-phosphate adenylyltransferase family protein |
| PG_005552_T.1 | 39 | 0.7 | 0.032 | 6 | AT1G30220 | inositol transporter 2 |
| PG_019871_T.1 | 29 | 0.7 | 0.047 | 6 | AT5G05010 | clathrin adaptor complexes medium subunit family protein |
| PG_002303_T.1 | 28 | 0.7 | 0.035 | 6 | AT3G12060 | Plant protein of unknown function (DUF828) |
| PG_029798_T.1 | 46 | 0.7 | 0.021 | 6 | N/A | N/A |
| PG_019923_T.1 | 66 | 0.7 | 0.027 | 6 | AT3G06350 | dehydroquinate dehydratase, putative / shikimate dehydrogenase, putative |
| PG_006850_T.1 | 41 | 0.7 | 0.018 | 6 | AT1G30220 | inositol transporter 2 |
| PG_014120_T.1 | 34 | 0.7 | 0.039 | 6 | AT5G53300 | ubiquitin-conjugating enzyme 10 |
| PG_012759_T.1 | 32 | 0.8 | 0.017 | 6 | AT5G50920 | CLPC homologue 1 |
| PG_001907_T.1 | 33 | 0.8 | 0.032 | 6 | AT4G02280 | sucrose synthase 3 |
| PG_031268_T.1 | 76 | 0.8 | 0.011 | 6 | AT1G77330 | 2-oxoglutarate (2OG) and Fe(II)-dependent oxygenase superfamily protein |
| PG_015103_T.1 | 51 | 0.8 | 0.030 | 6 | AT3G02540 | Rad23 UV excision repair protein family |
| PG_013577_T.1 | 101 | 0.8 | 0.044 | 4 | AT4G32000 | Protein kinase superfamily protein |
| PG_009280_T.1 | 36 | 0.8 | 0.029 | 6 | AT1G17420 | lipoxygenase 3 |
| PG_023005_T.1 | 72 | 0.8 | 0.010 | 6 | N/A | N/A |
| PG_005360_T.1 | 110 | 0.8 | 0.009 | 4 | AT5G03760 | Nucleotide-diphospho-sugar transferases superfamily protein |
| PG_025755_T.1 | 45 | 0.8 | 0.017 | 6 | N/A | N/A |
| PG_010495_T.1 | 44 | 0.8 | 0.023 | 6 | N/A | N/A |
| PG_005272_T.1 | 42 | 0.8 | 0.006 | 6 | AT5G51070 | Clp ATPase |
| PG_037304_T.1 | 26 | 0.8 | 0.029 | 6 | N/A | N/A |
| PG_006967_T.1 | 35 | 0.8 | 0.012 | 6 | N/A | N/A |
| PG_010547_T.1 | 63 | 0.8 | 0.045 | 6 | AT1G26910 | Ribosomal protein L16p/L10e family protein |
| PG_010130_T.1 | 80 | 0.8 | 0.004 | 6 | AT3G19450 | GroES-like zinc-binding alcohol dehydrogenase family protein |
| PG_006006_T.1 | 45 | 0.8 | 0.006 | 6 | AT1G56460 | HIT zinc finger ;PAPA-1-like conserved region |
| PG_007824_T.1 | 26 | 0.8 | 0.033 | 4 | AT5G67210 | Protein of unknown function (DUF579) |
| PG_014731_T.1 | 27 | 0.8 | 0.023 | 6 | N/A | N/A |
| PG_002557_T.1 | 169 | 0.8 | 0.025 | 6 | AT2G21660 | cold, circadian rhythm, and rna binding 2 |
| PG_013490_T.1 | 106 | 0.9 | 0.005 | 6 | AT1G71695 | Peroxidase superfamily protein |
| PG_007289_T.1 | 33 | 0.9 | 0.008 | 6 | AT3G46580 | methyl-CPG-binding domain protein 5 |
| PG_013827_T.1 | 47 | 0.9 | 0.020 | 6 | AT4G33510 | 3-deoxy-d-arabino-heptulosonate 7-phosphate synthase |
| PG_014372_T.1 | 25 | 0.9 | 0.008 | 6 | AT4G34200 | D-3-phosphoglycerate dehydrogenase |
| PG_011199_T.1 | 48 | 0.9 | 0.006 | 6 | AT1G60940 | SNF1-related protein kinase 2.10 |
| PG_008700_T.1 | 38 | 0.9 | 0.025 | 6 | AT1G15520 | pleiotropic drug resistance 12 |
| PG_001926_T.1 | 42 | 0.9 | 0.013 | 6 | AT1G76690 | 12-oxophytodienoate reductase 2 |
| PG_013468_T.1 | 39 | 0.9 | 0.009 | 4 | N/A | N/A |
| PG_027328_T.1 | 112 | 0.9 | 0.022 | 6 | N/A | N/A |
| PG_006779_T.1 | 34 | 0.9 | 0.020 | 4 | AT5G67230 | Nucleotide-diphospho-sugar transferases superfamily protein |
| PG_024659_T.1 | 40 | 0.9 | 0.002 | 4 | AT3G03780 | methionine synthase 2 |
| PG_022434_T.1 | 36 | 0.9 | 0.005 | 6 | AT2G44160 | methylenetetrahydrofolate reductase 2 |
| PG_026186_T.1 | 45 | 1.0 | 0.006 | 6 | AT4G11650 | osmotin 34 |
| PG_007018_T.1 | 48 | 1.0 | 0.027 | 6 | AT2G38800 | Plant calmodulin-binding protein-related |
| PG_023832_T.1 | 45 | 1.0 | 0.021 | 4 | N/A | N/A |
| PG_014701_T.1 | 30 | 1.0 | 0.007 | 6 | N/A | N/A |
| PG_016715_T.1 | 317 | 1.0 | 0.042 | 6 | N/A | N/A |
| PG_001634_T.1 | 64 | 1.0 | 0.032 | 6 | AT3G17390 | S-adenosylmethionine synthetase family protein |
| PG_014106_T.1 | 47 | 1.0 | 0.005 | 6 | AT3G52930 | Aldolase superfamily protein |
| PG_007721_T.1 | 142 | 1.0 | 0.021 | 6 | AT4G34050 | S-adenosyl-L-methionine-dependent methyltransferases superfamily protein |
| PG_004789_T.1 | 40 | 1.0 | 0.029 | 6 | AT5G46020 | unknown |
| PG_006581_T.1 | 24 | 1.0 | 0.045 | 6 | AT2G28110 | Exostosin family protein |
| PG_015725_T.1 | 33 | 1.0 | 0.009 | 6 | AT3G57880 | Calcium-dependent lipid-binding (CaLB domain) plant phosphoribosyltransferase family protein |
| PG_005525_T.1 | 100 | 1.0 | 0.009 | 4 | AT3G17390 | S-adenosylmethionine synthetase family protein |
| PG_011456_T.1 | 40 | 1.0 | 0.005 | 6 | AT5G26340 | Major facilitator superfamily protein |
| PG_016635_T.1 | 132 | 1.0 | 0.001 | 4 | AT2G44160 | methylenetetrahydrofolate reductase 2 |
| PG_014177_T.1 | 30 | 1.0 | 0.004 | 4 | AT2G30490 | cinnamate-4-hydroxylase |
| PG_004966_T.1 | 100 | 1.0 | 0.001 | 6 | AT2G16500 | arginine decarboxylase 1 |
| PG_005352_T.1 | 45 | 1.0 | 0.006 | 4 | AT5G03170 | FASCICLIN-like arabinogalactan-protein 11 |
| PG_013775_T.1 | 62 | 1.0 | 0.006 | 4 | AT5G15630 | COBRA-like extracellular glycosyl-phosphatidyl inositol-anchored protein family |
| PG_005378_T.1 | 81 | 1.0 | 0.002 | 4 | AT3G21240 | 4-coumarate:CoA ligase 2 |
| PG_005422_T.1 | 14 | 1.1 | 0.048 | 6 | AT4G39230 | NmrA-like negative transcriptional regulator family protein |
| PG_008642_T.1 | 62 | 1.1 | 0.001 | 6 | AT4G34350 | 4-hydroxy-3-methylbut-2-enyl diphosphate reductase |
| PG_022656_T.1 | 28 | 1.1 | 0.003 | 6 | AT5G49190 | sucrose synthase 2 |
| PG_010525_T.1 | 33 | 1.1 | 0.002 | 6 | AT4G35630 | phosphoserine aminotransferase |
| PG_020263_T.1 | 68 | 1.1 | 0.008 | 6 | AT4G02780 | Terpenoid cyclases/Protein prenyltransferases superfamily protein |
| PG_016174_T.1 | 24 | 1.1 | 0.010 | 6 | AT4G21320 | Aldolase-type TIM barrel family protein |
| PG_014385_T.1 | 18 | 1.2 | 0.012 | 6 | AT1G20160 | Subtilisin-like serine endopeptidase family protein |
| PG_021895_T.1 | 63 | 1.2 | 0.010 | 6 | AT2G24210 | terpene synthase 10 |
| PG_012056_T.1 | 49 | 1.2 | 2.5E-04 | 4 | AT1G08080 | alpha carbonic anhydrase 7 |
| PG_005410_T.1 | 14 | 1.2 | 0.039 | 6 | AT3G06260 | galacturonosyltransferase-like 4 |
| PG_011298_T.1 | 26 | 1.3 | 0.006 | 4 | AT4G16260 | Glycosyl hydrolase superfamily protein |
| PG_019918_T.1 | 31 | 1.3 | 0.001 | 6 | AT5G43830 | Aluminium induced protein with YGL and LRDR motifs |
| PG_010124_T.1 | 18 | 1.3 | 0.018 | 5 | AT4G16730 | terpene synthase 02 |
| PG_000043_T.1 | 46 | 1.3 | 1.2E-04 | 4 | AT5G08370 | alpha-galactosidase 2 |
| PG_033664_T.1 | 64 | 1.3 | 0.001 | 6 | AT4G25000 | alpha-amylase-like |
| PG_000261_T.1 | 24 | 1.3 | 0.001 | 6 | AT3G03780 | methionine synthase 2 |
| PG_015664_T.1 | 38 | 1.4 | 0.001 | 6 | AT1G17420 | lipoxygenase 3 |
| PG_005268_T.1 | 18 | 1.4 | 0.025 | 4 | N/A | N/A |
| PG_007184_T.1 | 47 | 1.4 | 0.004 | 5 | AT4G16730 | terpene synthase 02 |
| PG_013849_T.1 | 24 | 1.5 | 0.004 | 5 | AT4G02780 | Terpenoid cyclases/Protein prenyltransferases superfamily protein |
| PG_001750_T.1 | 38 | 1.6 | 0.002 | 6 | AT3G25810 | Terpenoid cyclases/Protein prenyltransferases superfamily protein |
| PG_009311_T.1 | 19 | 1.6 | 0.003 | 6 | N/A | N/A |
| PG_011104_T.1 | 88 | 1.6 | 0.001 | 5 | AT4G36220 | ferulic acid 5-hydroxylase 1 |
| PG_005297_T.1 | 27 | 1.7 | 1.6E-05 | 4 | AT5G17920 | Cobalamin-independent synthase family protein |
| PG_026381_T.1 | 48 | 1.7 | 0.019 | 3 | N/A | N/A |
| PG_021633_T.1 | 56 | 1.7 | 2.3E-06 | 6 | AT3G22142 | Bifunctional inhibitor/lipid-transfer protein/seed storage 2S albumin superfamily protein |
| PG_000042_T.1 | 16 | 1.7 | 0.004 | 6 | AT5G08380 | alpha-galactosidase 1 |
| PG_012543_T.1 | 35 | 1.8 | 2.0E-05 | 4 | AT1G20450 | Dehydrin family protein |
| PG_005984_T.1 | 28 | 1.8 | 1.6E-04 | 4 | AT5G08370 | alpha-galactosidase 2 |
| PG_023690_T.1 | 45 | 1.9 | 0.016 | 3 | N/A | N/A |
| PG_025920_T.1 | 20 | 1.9 | 1.4E-04 | 6 | AT5G04080 | unknown |
| PG_007975_T.1 | 33 | 1.9 | 0.001 | 6 | AT4G16740 | terpene synthase 03 |
| PG_014088_T.1 | 108 | 2.0 | 4.2E-05 | 5 | AT5G54160 | O-methyltransferase 1 |
| PG_001208_T.1 | 30 | 2.1 | 0.003 | 3 | AT5G02500 | heat shock cognate protein 70-1 |
| PG_021367_T.1 | 24 | 2.1 | 8.1E-07 | 6 | N/A | N/A |
| PG_016491_T.1 | 446 | 2.2 | 0.015 | 3 | N/A | N/A |
| PG_007852_T.1 | 30 | 2.2 | 1.2E-05 | 6 | AT1G75280 | NmrA-like negative transcriptional regulator family protein |
| PG_026593_T.1 | 31 | 2.2 | 0.001 | 3 | N/A | N/A |
| PG_022114_T.1 | 120 | 2.2 | 0.006 | 3 | N/A | N/A |
| PG_024204_T.1 | 43 | 2.3 | 3.2E-04 | 3 | N/A | N/A |
| PG_007447_T.1 | 39 | 2.3 | 3.7E-09 | 6 | AT4G33300 | ADR1-like 1 |
| PG_011484_T.1 | 286 | 2.3 | 0.016 | 3 | AT2G46240 | BCL-2-associated athanogene 6 |
| PG_011735_T.1 | 867 | 2.4 | 0.027 | 5 | AT1G26560 | beta glucosidase 40 |
| PG_022805_T.1 | 52 | 2.4 | 0.001 | 3 | N/A | N/A |
| PG_025131_T.1 | 79 | 2.4 | 3.4E-04 | 3 | AT1G12060 | BCL-2-associated athanogene 5 |
| PG_022452_T.1 | 91 | 2.4 | 0.001 | 3 | AT1G12060 | BCL-2-associated athanogene 5 |
| PG_003371_T.1 | 483 | 2.5 | 0.006 | 3 | N/A | N/A |
| PG_022967_T.1 | 73 | 2.5 | 0.038 | 3 | N/A | N/A |
| PG_014125_T.1 | 34 | 2.6 | 2.3E-09 | 6 | AT3G54420 | homolog of carrot EP3-3 chitinase |
| PG_012797_T.1 | 25 | 2.6 | 1.6E-06 | 3 | AT5G52640 | heat shock protein 90.1 |
| PG_001513_T.1 | 128 | 2.6 | 3.2E-04 | 3 | N/A | N/A |
| PG_022569_T.1 | 139 | 2.7 | 0.001 | 3 | N/A | N/A |
| PG_024016_T.1 | 70 | 2.7 | 5.3E-05 | 3 | N/A | N/A |
| PG_035827_T.1 | 22 | 2.7 | 3.9E-07 | 6 | AT3G54420 | homolog of carrot EP3-3 chitinase |
| PG_004462_T.1 | 23 | 2.7 | 6.2E-05 | 3 | AT4G11600 | glutathione peroxidase 6 |
| PG_007585_T.1 | 18 | 3.9 | 1.7E-10 | 6 | AT2G43590 | Chitinase family protein |
| PG_021717_T.1 | 21 | 4.1 | 2.1E-13 | 6 | AT2G32300 | uclacyanin 1 |
| PG_023444_T.1 | 31 | 4.6 | 2.0E-07 | 3 | AT4G27670 | heat shock protein 21 |

### Table S5

Gene enrichment analysis using gene ontology (GO) for metabolic functions and biological processes and pathways of Kyoto Encyclopedia Gene and Genome (KEGG). Clusters are gene groups identified with the Gap PAM statistical approach (Fig. S5). The cluster number represents the top to bottom order of the phylogeny. The numbers in parenthesis next to the cluster indicate the total number of genes for the analysis. Gene, fold and Benjamini indicate the number of gene associated to the term/category, the fold enrichment, and the corrected P-value, respectively. This analysis was done using DAVID with a background list of 22532 annotated *Picea glauca* genes from the reference transcriptome.

| Category | Term | Gene | Fold | Benjamini |
| --- | --- | --- | --- | --- |
| *GO term* | *Cluster 3 (5)* |  |  |  |
|  | GO:0051087~chaperone binding | 2 | 269 | 0.041 |
|  | GO:0009408~response to heat | 4 | 93 | <0.001 |
|  | *Cluster 4 (19)* |  |  |  |
|  | GO:0009834~plant-type secondary cell wall biogenesis | 5 | 111 | <0.001 |
|  | GO:0006730~one-carbon metabolic process | 3 | 86 | 0.018 |
|  | *Cluster 6 (77)* |  |  |  |
|  | GO:0034768~(E)-beta-ocimene synthase activity | 3 | 118 | 0.019 |
|  | GO:0050551~myrcene synthase activity | 3 | 142 | 0.025 |
| *KEGG pathway* | *Cluster 1 (4)* |  |  |  |
|  | ath04141:protein processing in endoplasmic reticulum | 3 | 16 | 0.036 |
|  | *Cluster 5 (4)* |  |  |  |
|  | ath00940:phenylpropanoid biosynthesis | 3 | 23 | 0.019 |
|  | ath01110:biosynthesis of secondary metabolites | 4 | 4 | 0.034 |
|  | *Cluster 6 (48)* |  |  |  |
|  | ath01110:biosynthesis of secondary metabolites | 24 | 2 | 0.003 |
|  | ath01130:biosynthesis of antibiotics | 13 | 3 | 0.016 |
|  | ath01230:biosynthesis of amino acids | 10 | 4 | 0.018 |
|  | ath01100:metabolic pathways | 30 | 2 | 0.022 |
|  | ath01200:carbon metabolism | 9 | 3 | 0.038 |

### Fig. S1

Resistance biomarker levels as a function of phenological stage in *Picea glauca* during 2016. Trees of twenty different genotypes classified as resistant (R, n = 11) and susceptible (S, n = 9) were evaluated in a mismatched (early) and matched (synchronized) phenological window of defense. Analysis of variance type III for intra-annual variation of current year foliage of R and S trees. R trees: picein F = 4.70, P = 0.0002; piceol F = 2.33, P = 0.0347; pungenol F = 3.77, P = 0.0018. S trees: picein F = 13.30, P < 0.0001; piceol F = 0.91, P = 0.5065; pungenol F = 1.97, P = 0.0740. Letters indicate significant differences between time points in the seasonal phenology according to Tukey multiple comparison test (α = 0.05).

### Fig. S2

Correlation between levels of *Pgβglu-1* transcripts using gene-specific primers and RT-qPCR or RNA-Seq.


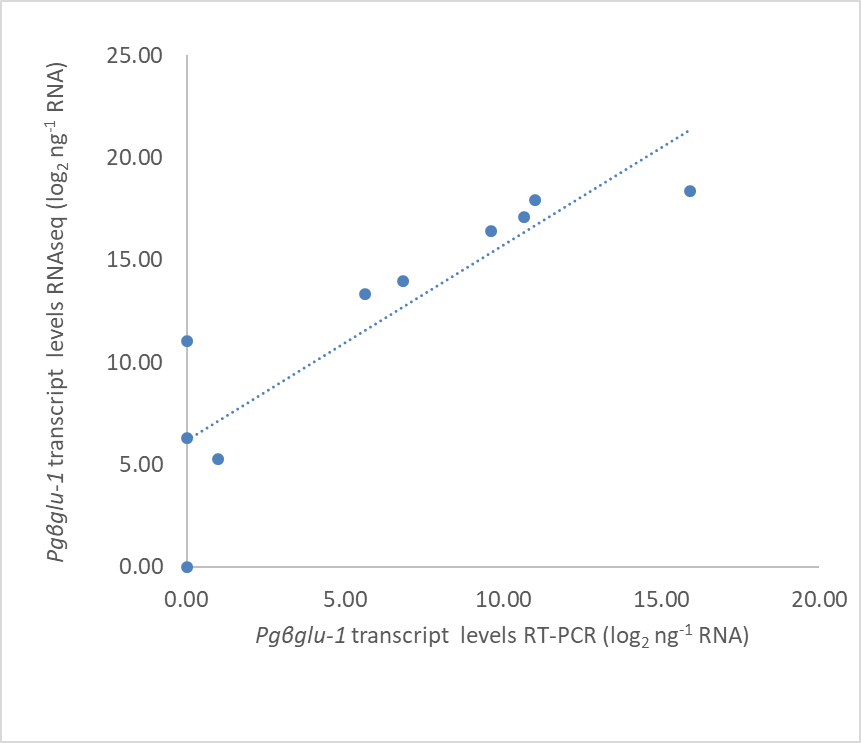


### Fig. S3

Variability of RNA-Seq library sizes (millions of reads) for the induction of the defense mechanism of *Picea glauca* in the current year foliage. Trees were control or treated with *Choristoneura fumiferana* (SBW).


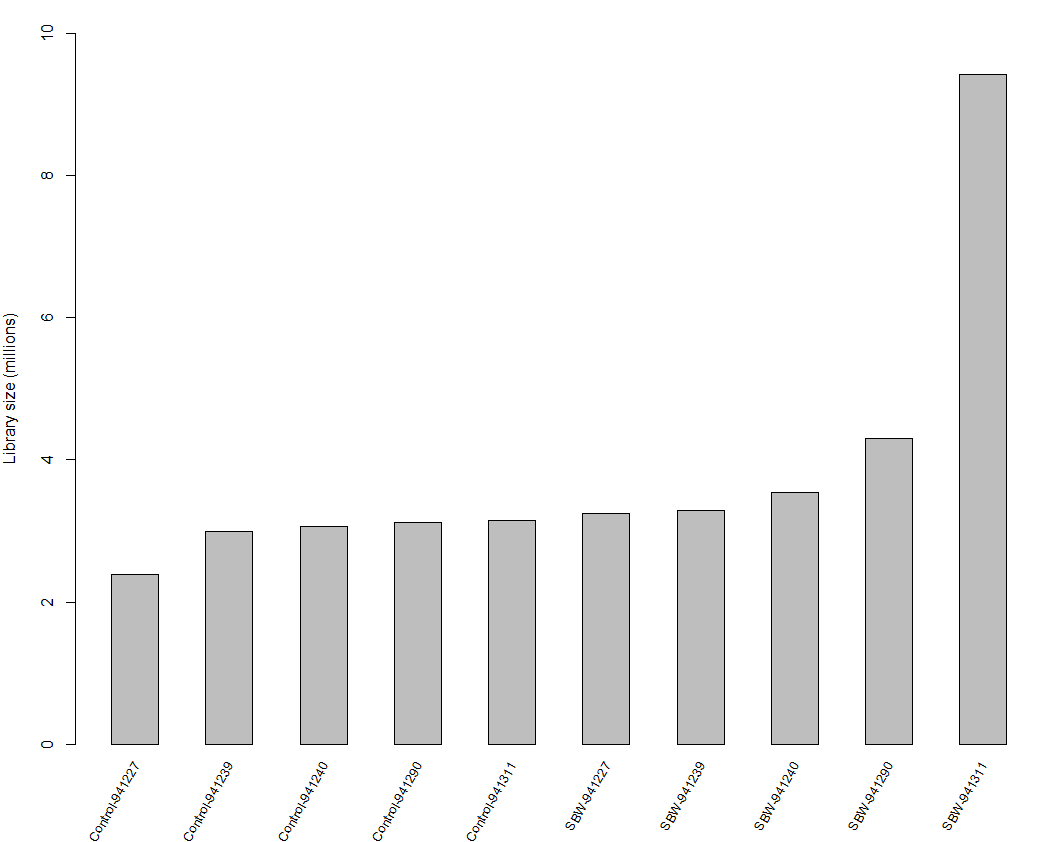


### Fig. S4

Gene expression before a) and after b) normalization for genes with the highest levels of transcripts (counts) during the induction of the defense mechanism of *Picea glauca*. Differentially expressed genes in the current year foliage between control and treated with *Choristoneura fumiferana* (SBW) trees. There are five genotypes with high (941240, 941290: resistant, dark green) or low levels of hydroxyacetophenone aglycons (941227, 941239, 941311: susceptible, pale green). Levels of gene expression are presented on a log_2_ scale.


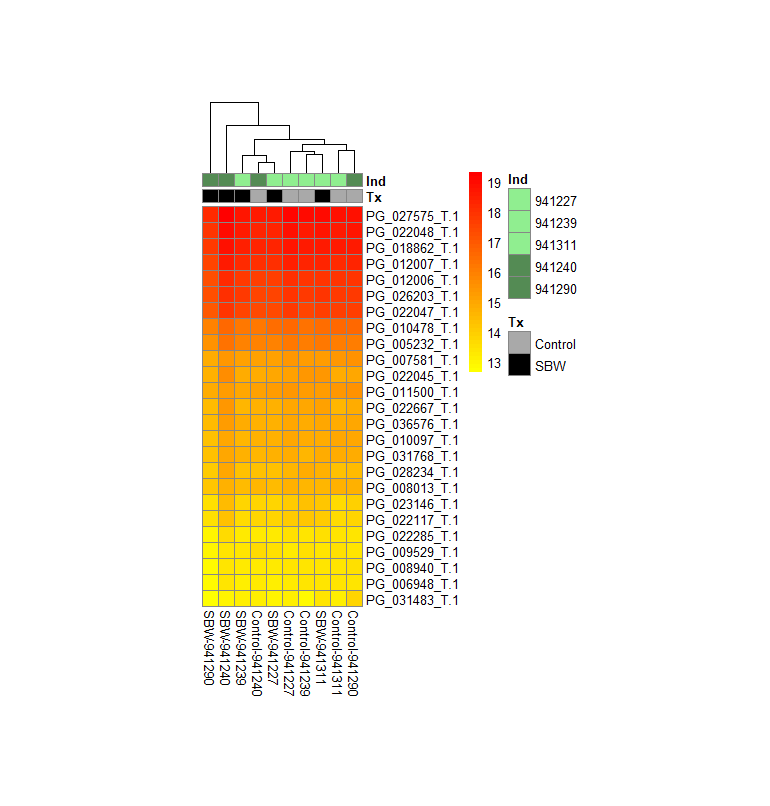


b)

a)


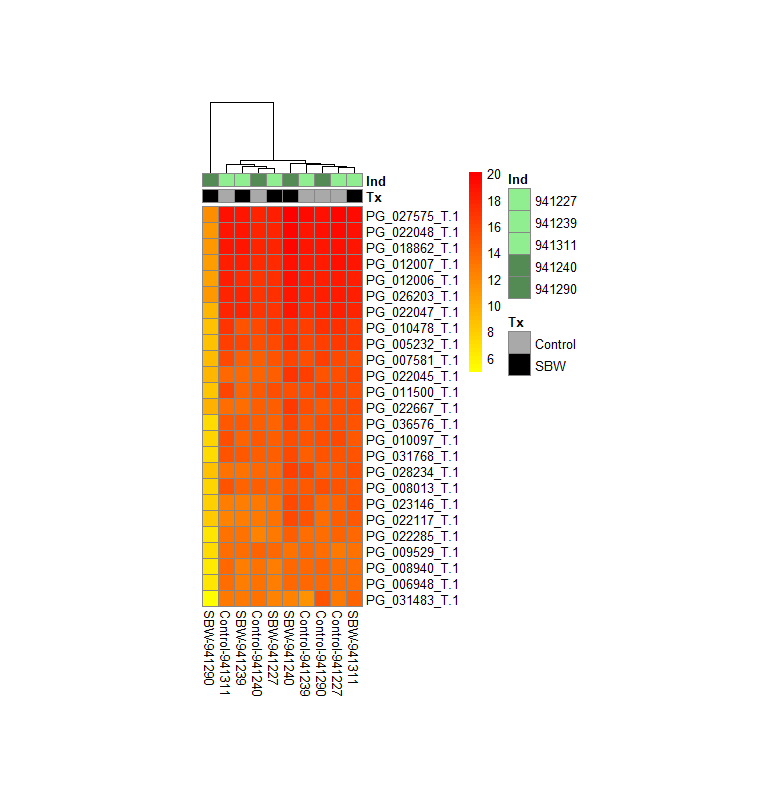


### Fig. S5

Determination of the optimal number of cluster of a clustering analysis. We used the factoextra v1.0.5 package with the Gap statistic analysis using partitioning around medoids, a robust version of K-means.


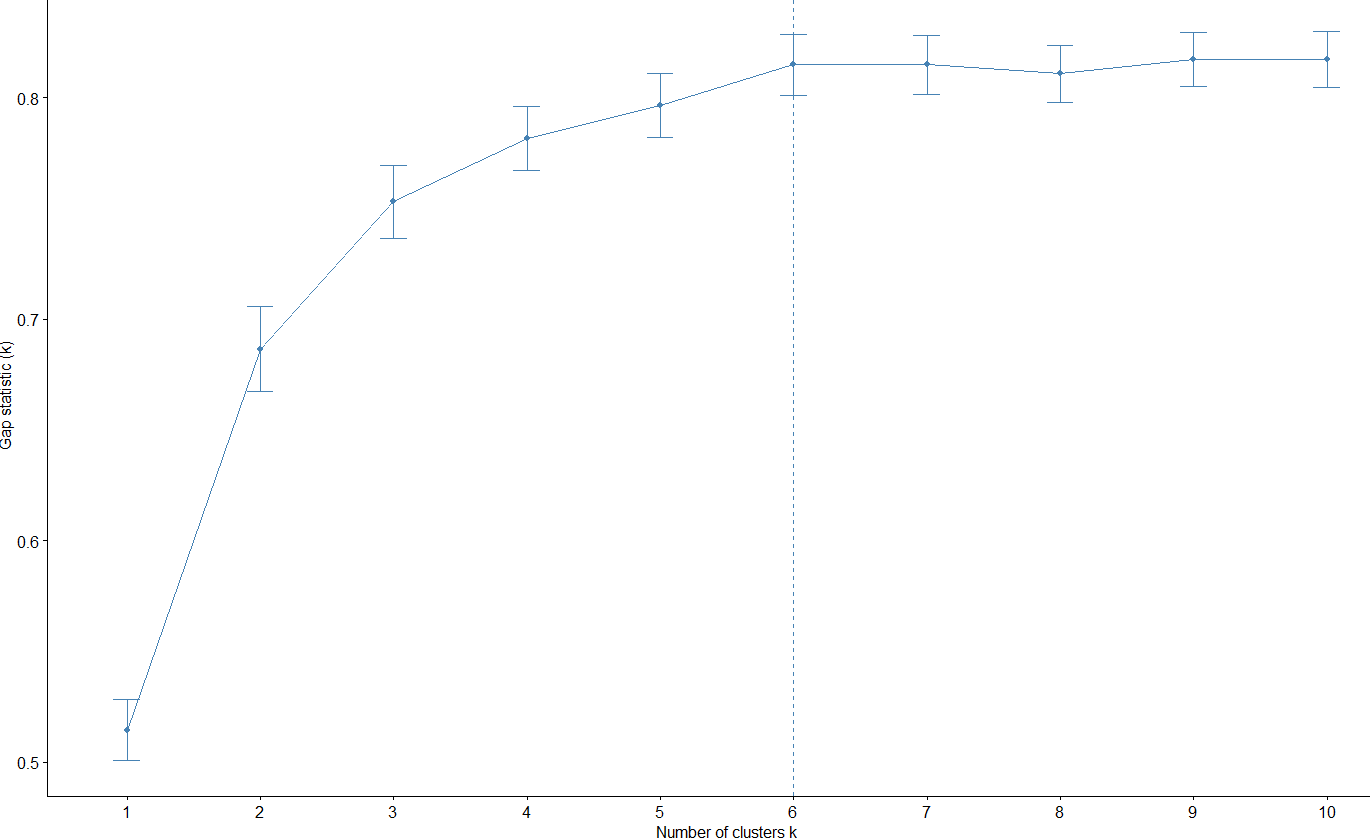


## Methods

### Field experiments

Two experiments were carried in a clonal bank planted a common garden established in 1999 in Valcartier, Quebec, Canada (46°56’N, 71°29’W) with several grafted replicates of each genotype.

### Experiment 1. Mismatch and match of tree resistance window

A total of 20 genotypes of *P. glauca* were used to investigate the effect of mismatch and match of the tree resistance phenology on spruce budworm. The experiment involved insect rearing in the field by using second instar larvae (L2) of *Choristoneura fumiferana* obtained from the Canadian Forest Service, (Sault Ste. Marie, Canada). The larvae were transferred to artificial diet (McMorran, 1965) three to four days before their installation on host trees; this was to activate their emergence from diapause. For the mismatched treatment and associated controls started on May 25^th^ and ended on June 30^th^; the matched treatment and associated controls started on June 15^th^ and ended on July 19^th^. The treatments consisted of placing 20 L2 on each of the two 40 to 70 cm long branches per tree in the middle third of the crown and then the branches were enclosed in fine-mesh bags (Bauce et al., 1994), which were visually examined weekly.

At the end of each treatment, the branches with *C. fumiferana* were removed to collect the pupae/larvae and determine the level of defoliation, insect mass, and sex. All of the recovered insects were individually transferred to cups with or without artificial diet (larvae and pupae, respectively) and kept in a growing chamber at 23° C and a 16h/8h photoperiod to estimate the developmental time (moment of butterfly emergence) and survival. In addition, the foliage was sampled from all of the trees on May 25, June 1, 8, 15, 21 and 30; July 5 and 19, 2016.

### Experiment 2. Induction with spruce budworm experiment with mature white spruce

We selected five unrelated *P. glauca* genotypes (941227, 941239, 941240, 941290, 941311) for their overall variability in piceol and pungenol content based on results presented in Mageroy and coll. (2015). On June 10^th^ 2015, four to six L2 of *C. fumiferana* (Canadian Forest Service, Sault Ste. Marie) were deposited on the year foliage of 3 different branches and covered by fine mesh bags of 1 m in length. Branches were located in the lower third of the trees. On June 29^th^ 2015, current year foliage samples were collected inside the bags where damage by the insect was visible on the treatment trees (15 samples) and control trees (without insects, 5 samples) of the same genotype (total 20 samples).

### Foliage conservation

The foliage samples were frozen in liquid nitrogen immediately after removal from the trees and stored at −80 °C. The needles were ground to powder using a MixerMill 300 (Retsch, <http://www.retsch.com/>) and steel grinding balls cooled in nitrogen. Powdered foliage tissue was stored at −80 °C until RNA extraction.

### RNA extraction and cDNA synthesis

RNA was extracted by grinding tissues in liquid nitrogen to a fine powder and by utilizing either (i) the cetyltrimethyl ammonium bromide extraction method as described by Chang et al. (1993) with modifications (Pavy et al., 2008). The total RNA concentration was determined using a NanoDrop 1000 (Thermo Scientific, <http://www.thermoscientific.com/>) and assessed for quality with an Agilent 2100 Bioanalyzer using High Sensitivity DNA chips (Agilent Technologies Inc., Santa Clara, CA, USA), and stored at −80 °C. Complementary DNAs were prepared from 500 ng of total RNA using Quantitect Reverse Transcription Kit (Qiagen, Germantown, MD, USA) and then diluted 1:4 in RNase-free water.

### RNA-Seq library synthesis

We used 500 ng of RNA per sample to synthetize mRNA libraries with TruSeq® Stranded mRNA kit (Illumina Canada Inc., Victoria, BC, Canada), following the manufacturer’s protocol with a few modifications. First, Illumina technology-suitable custom adapters were synthetized (IDT, Coralville, IA, USA) and their concentration was reduced by half at 25 nM per reaction to avoid adapter dimer molecules and Tris-NaCl (10 mM, 50 mM) at 25 nM was used to complete the volume. Second strand synthesis and marking clean-up and library amplification clean-up were performed using Axygen® AxyPrep™ Mag PCR Clean-Up Kit (Axygen Biosciences, Union City, CA, USA) whereas post ligation clean-up was done using a ratio of 0.85 of PEG/NaCl SPRI® Solution over Beads with adapter-ligated DNA. Distribution of library fragment size and the absence of dimer molecules were verified with an Agilent Bioanalyzer 2100 using High Sensitivity DNA chips (Agilent Technologies Inc., Santa Clara, CA, USA). DNA quantification was performed using Quant-iT™ PicoGreen ® dsDNA Reagent and Kits (Molecular Probes Inc., Eugene, OR, USA). Two equimolar pools of 16 libraries each (22 samples not used in this study) were prepared. Each of the pools were sequenced with an Illumina HiSeq 2500 with paired-end 2 x 250bp technology with one pool per lane at the Genome Quebec Innovation Centre at McGill University (Montreal., Quebec, Canada).

### Read assembly and annotation

Raw RNA-Seq sequence (FASTQ) files were filtered into High-Quality (HQ) sequences, trimming adapters using the NUCLEAR version 3.2.4 software (Gydle Inc.) and retaining only segments of 50 consecutive Q20+ bases. The HQ sequences were mapped to ribosomal and gene sequences using the *P. glauca* Gene catalogue (Rigault et al., 2011) using NUCLEAR. A total of 22532 gene sequences (i.e. cluster representative clone sequences) were annotated by performing protein-level similarity searches with BLASTX (E-value # 1e-10) against proteins of Arabidopsis (Arabidopsis thaliana; The Arabidopsis Information Resource [TAIR] version 10) (see Rigault et al., 2011 for more details).

### Gene expression analysis

Count data were normalized with DEseq2 v1.20.0 package in R software (Core Team 2018) . The size of one library was five times larger than the smallest library (Fig. S3). After normalization, expression across library was similar for the highest and lowest expressed genes (Fig. S4). Since our sample size is small, we decided to add a filtration step to remove genes with low expression; only genes expressed in two libraries at levels greater or equal to 50 counts were kept for subsequent analyses. We calculated the differential expression between the treatment and the control foliage samples using genotype as a nested factor with the DEseq2 v1.20.0 package. Only genes with P < 0.05 and fold change in expression greater than 0.5 (absolute) were considered for further analysis. We did not used the Benjamini adjusted p values since they seem too conservative and favor type II errors. We detected a significant increase in the *Pgβglu-1* transcript levels with Reverse transcription-qPCR (RT-qPCR)(see below), and included the results in the list of differentially expressed genes with the unadjusted p value (Table. S4). Cluster v2.0.7., factoextra v1.0.5, and pheatmap v1.0.10 packages were used to generate a heatmap with a gene cluster analyses with manhattan distances and ward.D2 clustering method.

### Gene enrichment analysis

DAVID (database for annotation, visualization and integrated discovery) version 6.8 functional annotation tool (Huang et al., 2009a; 2009b) was used to identify enriched gene ontology metabolic functions and biological processes, pathways of Kyoto Encyclopedia Gene and Genome (KEGG) and protein sequence classification (InterPro). We used for background gene list the 10832 different annotations of the 22 532 *P. glauca* genes. We used the medium stringency for gene classification in DAVID and used the Benjamini corrected P value (Benjamini & Hochberg, 1995) to identify significantly enriched terms.

### Reverse transcriptase-qPCR analysis

We used RT-qPCR with *Pgβglu-1* gene-specific primers (Mageroy et al., 2015) to validate RNAseq library results. The PCR mixtures were composed with a QuantiFast^®^SYBR^®^ Green PCR kit (Qiagen) as follows: 1x master mix, 300 nM of 5’ and 3’ primers and 5 µl of cDNA in a final volume of 15 µl. Amplifications were carried out in a LightCycler^®^480 (Roche, <http://www.roche.com/>) as described in Boyle et al. (2009). The LRE method (Rutledge & Stewart 2008) adapted for Excel (Boyle et al., 2009) was used to calculate the number of transcript molecules, which was normalized to a ratio calculated by the geometric mean of three reference genes: elongation factor 1a (EF1-a) (BT102965), cell division cycle 2 (CDC2) (BT106071) and ribosomal protein L3A (BT115036) as described elsewhere (Beaulieu et al., 2013). Levels of *Pgβglu-1* transcripts measured with RNA-Seq were correlated with the RT-qPCR data (Fig. S2, Pearson correlation = 0.88, P < 0.001).

### Extraction and quantification of acetophenone compounds

Acetophenones were extracted as described in Mageroy et al. (2015). Picein, piceol and pungenol were identified and quantified by LC (Agilent 1200 series) coupled to a MS detector (Agilent 6210 TOF) as described in Parent et al. (2017). Quantification was performed using external calibration curves.

## References

Bauce, E., & Kumbasli, M. 2007. Natural resistance of fast growing white spruce, *Picea glauca* (Moench), trees against spruce budworm, *Choristoneura fumiferana* (Clem.). In Bottlenecks, Solutions, and Priorities in the Context of Functions of Forest Resources: Proceedings of the International Symposium, Istanbul, Turkey, 17–19 October 2007. Edited by M. Demir and E. Yilmaz. Tubitak Istanbul, Ankara, Turkey, 687–695.

Bauce, E., Crépin, M., & Carisey, N. (1994). Spruce budworm growth, development and food utilization on young and old balsam fir trees. Oecologia, 97, 499-507.

Beaulieu, J., Doerksen, T. K., MacKay, J., Rainville, A., & Bousquet, J. (2014). Genomic selection accuracies within and between environments and small breeding groups in white spruce. BMC Genomics, 15, 1048.

Benjamini, Y., & Hochberg, Y. (1995). Controlling the false discovery rate: a practical and powerful approach to multiple testing. Journal of the Royal Statistical Society, 57, 289-300.

Boyle, B., Dallaire, N., & MacKay, J. (2009). Evaluation of the impact of single nucleotide polymorphisms and primer mismatches on quantitative PCR. BMC Biotechnology, 9, 75.

Chang, S., Puryear, J., & Cairney, J. (1993). A simple and efficient method for isolating RNA from pine trees. Plant Molecular Biology Reporter, 11, 113–116.

Huang, D. W., Sherman, B. T., & Lempicki, R. A. (2009). Systematic and integrative analysis of large gene lists using DAVID bioinformatics resources. Nature Protocols, 4, 44-57.

Huang, D. W., Sherman, B. T., & Lempicki, R. A. (2009). Bioinformatics enrichment tools: Paths toward the comprehensive functional analysis of large gene lists. Nucleic Acids Research, 37, 1–13.

Love, M. I., Huber, W., & Anders, S. (2014). Moderated estimation of fold change and dispersion for RNA-seq data with DESeq2. Genome Biology, 15, 550.

Mageroy, M. H., Parent, G., Germanos, G., Giguère, I., Delvas, N., Maaroufi, H., … Mackay, J. J. (2015). Expression of the β-glucosidase gene *Pgβglu-1* underpins natural resistance of white spruce against spruce budworm. Plant Journal., 81, 68–80.

McMorran, A. (1965). A synthetic diet for the spruce budworm, *Choristoneura fumiferana*. The Canadian Entomologist, 97, 58–62.

Parent, G. J., Giguère, I., Germanos, G., Lamara, M., Bauce, É., & Mackay, J. J. (2017). Insect herbivory (*Choristoneura fumiferana*, Tortricidea) underlies tree population structure (*Picea glauca*, Pinaceae). Scientific Reports, 7, 42273.

Pavy, N., Boyle, B., Nelson, C., Paule, C., Giguère, I., Caron, S., … Mackay, J. (2008). Identification of conserved core xylem gene sets: conifer cDNA microarray development, transcript profiling and computational analyses. New Phytologist, 180, 766–786.

Rigault, P., Boyle, B., Lepage, P., Cooke, J. E. K., Bousquet, J., & MacKay, J. J. (2011). A white spruce gene catalog for conifer genome analyses. Plant Physiology, 157, 14–28.

Rutledge, R. G., & Stewart, D. (2008). A kinetic-based sigmoidal model for the polymerase chain reaction and its application to high-capacity absolute quantitative real-time PCR. BMC Biotechnology, 8, 47.

Team, R. D. C., & R Development Core Team, R. (2018). R: a language and environment for statistical computing. *R Foundation for Statistical Computing*. Vienna: Austria: R Foundation for Statistical Computing.
